# Impacts of fire and prospects for recovery in a tropical peat forest ecosystem

**DOI:** 10.1073/pnas.2307216121

**Published:** 2024-04-15

**Authors:** Mark E. Harrison, Nicolas J. Deere, Muhammad Ali Imron, Darmae Nasir, Adul, Hastin Ambar Asti, Joana Aragay Soler, Nicholas C. Boyd, Susan M. Cheyne, Sarah A. Collins, Laura J. D’Arcy, Wendy M. Erb, Hannah Green, William Healy, Hendri, Brendan Holly, Peter R. Houlihan, Simon J. Husson, Iwan, Karen A. Jeffers, Ici P. Kulu, Kitso Kusin, Nicholas C. Marchant, Helen C. Morrogh-Bernard, Susan E. Page, Ari Purwanto, Bernat Ripoll Capilla, Oscar Rodriguez de Rivera Ortega, Santiano, Katie L. Spencer, Jito Sugardjito, Jatna Supriatna, Sara A. Thornton, F. J. Frank van Veen, Yulintine, Matthew J. Struebig

**Affiliations:** ^a^Centre for Ecology and Conservation, Faculty of Environment, Science and Economy, University of Exeter, Penryn TR10 9FE, United Kingdom; ^b^School of Geography, Geology and the Environment, University of Leicester, Leicester LE1 7RH, United Kingdom; ^c^Durrell Institute of Conservation and Ecology, School of Anthropology and Conservation, University of Kent, Canterbury CT2 7NR, United Kingdom; ^d^Faculty of Forestry, Universitas Gadjah Mada, Yogyakarta 55281, Indonesia; ^e^Centre for the International Cooperation in Sustainable Management of Tropical Peatlands, University of Palangka Raya, Palangka Raya 73112, Central Kalimantan, Indonesia; ^f^Yayasan Borneo Nature Indonesia, Palangka Raya 73112, Central Kalimantan, Indonesia; ^g^Wildlife Conservation Research Unit, Department of Biology, University of Oxford, Oxford OX13 5QL, United Kingdom; ^h^Department of Modern Languages, University of Wales Aberystwyth, Aberystwth SY23 1DE, United Kingdom; ^i^School of Humanities and Social Sciences, Oxford Brookes University, Oxford OX3 0BP, United Kingdom; ^j^School of Biological and Marine Sciences, Faculty of Science and Engineering, University of Plymouth, Plymouth PL4 8AA, United Kingdom; ^k^Borneo Nature Foundation International, Tremough Innovation Centre, Penryn TR10 9TA, United Kingdom; ^l^K. Lisa Yang Center for Conservation Bioacoustics, Cornell Lab of Ornithology, Cornell University, Ithaca, NY 14850; ^m^Environmental Studies, Centre College, Danville, KY 40422; ^n^Center for Tropical Research, Institute of the Environment and Sustainability, University of California, Los Angeles, Los Angeles, CA 90095-1496; ^o^Department of Mathematics and Statistics, Faculty of Environment, Science and Economy, University of Exeter, Exeter EX4 4QF, United Kingdom; ^p^Centre for Sustainable Energy and Resources Management, Universitas Nasional, Jakarta 12520, Indonesia; ^q^Faculty of Biology, Universitas Nasional, Jakarta 12520, Indonesia; ^r^Department of Biology, Faculty of Mathematics and Natural Sciences, Universitas Indonesia, Depok 16424, Indonesia

**Keywords:** biodiversity, fire regime, megafire, multi-taxon, restoration

## Abstract

Uncontrolled fires place considerable burdens on forest ecosystems, compromising our ability to meet conservation and restoration goals. A poor understanding of the impacts of fire on ecosystems and their biodiversity exacerbates this challenge, particularly in tropical regions where few studies have applied consistent analytical techniques to examine a broad range of ecological impacts over multiyear time frames. We compiled 16 y of data on ecosystem properties (17 variables) and biodiversity (21 variables) from a tropical peatland in Indonesia to assess fire impacts and infer the potential for recovery. Burned forest experienced altered structural and microclimatic conditions, resulting in a proliferation of nonforest vegetation and erosion of forest ecosystem properties and biodiversity. Compared to unburned forest, habitat structure, tree density, and canopy cover deteriorated by 58 to 98%, while declines in species diversity and abundance were most pronounced for trees, damselflies, and butterflies, particularly for forest specialist species. Tracking ecosystem property and biodiversity datasets over time revealed most to be sensitive to recurrent high-intensity fires within the wider landscape. These megafires immediately compromised water quality and tree reproductive phenology, crashing commercially valuable fish populations within 3 mo and driving a gradual decline in threatened vertebrates over 9 mo. Burned forest remained structurally compromised long after a burn event, but vegetation showed some signs of recovery over a 12-y period. Our findings demonstrate that, if left uncontrolled, fire may be a pervasive threat to the ecological functioning of tropical forests, underscoring the importance of fire prevention and long-term restoration efforts, as exemplified in Indonesia.

## Significance

Fire management in tropical forests requires an understanding of the ecological impacts of burn events and the ecosystem’s capacity to recover. We investigate this by tracking multiple ecosystem properties and biodiversity variables over 16 y in a tropical peatland in Indonesia. Compared to unburned areas, burned forest contained fewer trees, was more open and hotter, with more nonforest vegetation, leading to reduced biodiversity. Tracking ecological variables in nonburned forest over time revealed the ecosystem’s sensitivity to recurrent, high-intensity fire within the wider landscape. Some recovery was evident in burned areas within 12 y, but repeated fire risks reversing this trend. While fire prevention is crucial, long-term, context-specific tropical forest restoration is needed to deal with the consequences of fire.

Fire is a powerful biological filter, influencing the successional dynamics of terrestrial ecosystems and the distribution of wildlife (1, 2). However, environmental change driven by anthropogenic activities disrupts natural fire regimes across the world, increasing the prevalence and impacts of fire (3). In particular, large-scale “megafires” are a global phenomenon causing major ecological disruption (4). Fire accounts for 41% of tropical forest loss globally (5), and at least 1,071 species across nine taxonomic groups are reported as threatened by altered fire regimes (6). Most of our understanding of the ecological impacts of fire comes from naturally fire-prone habitats (e.g., savannahs), with limited information available from humid tropical regions, which tend to comprise fire-sensitive ecosystems (7, 8). With global fire activity and impacts projected to increase alongside changes in climate and land use (3, 9), detailed insights into ecosystem-scale responses to fire are urgently needed to help safeguard the ecological integrity of fire-affected tropical biomes and prevent species extinctions.

The impacts of burning are most pronounced in ecosystems where fire is naturally rare (10). Tropical forests are particularly maladapted to tolerate and recover from fire-related damage, which impacts ecosystem functioning, regeneration dynamics, and carbon emissions (11, 12). Fire-induced tree mortality causes a marked change in forest structure (13, 14), facilitating compositional transitions that favor herbaceous vegetation and disturbance-tolerant pioneer species (15, 16). Such structural and compositional shifts can alter microclimatic conditions and increase the prevalence of flammable vegetation, leaving the ecosystem susceptible to recurrent fire (11). Fires can also cause extensive wildlife mortality and other health and behavioral impacts due to immolation, radiant heat, and toxic particulate inhalation (17–19). Subsequent deterioration or loss of vertebrate-mediated ecological processes may then influence postfire vegetation recovery (20), amplifying a feedback loop that compromises the future of the forest ecosystem. While current evidence indicates that ecological responses to fire are intricately linked, it is challenging to draw parallels among multiple studies while accounting for confounding effects of study design, geographic location, and disturbance legacies. Ecosystem-scale syntheses that better control for these confounding effects are necessary if we are to reliably compare fire impacts across multiple ecological components and infer interactions between fire, vegetation dynamics, and biodiversity.

The characteristics of the fire regime govern the recovery potential of burned ecosystems (21). Fire intensity underpins the magnitude of ecological damage, while fire frequency, within and between fire seasons, determines fire recurrence in a given area. Collectively, these characteristics dictate an ecosystem’s capacity to resist change and return to its predisturbance state (21). Recurrent, high-intensity fires thus threaten ecosystem stability and increase the risk of irreversible state shifts (22), with potentially grave consequences for biodiversity (6, 20). While proximity to a fire determines much of its impact, indirect consequences of fire, primarily via smoke or haze exposure, can extend the footprint of disturbance far beyond the burn extent (23).

The essential longitudinal data needed to track ecological trends relative to fire regimes are so far missing from appraisals of fire in tropical ecosystems. To date, most ecological assessments have compared properties of burned and unburned areas but have not examined both spatial and temporal variability in fire regimes (24). Such insights are particularly lacking for tropical peatlands, such as those in Indonesia, which are highly valued for their globally significant carbon stocks and biodiversity, but have become increasingly susceptible to fire in recent decades due to changing climatic conditions and land-use practices (25). While the impacts of tropical peatland fires on carbon emissions, public health, local communities, and the economy are well documented (26, 27), their ecological impacts remain relatively understudied, as does the ability of the ecosystem to regenerate naturally following fire (though see, e.g., ref. 28).

**Fig. 1.**
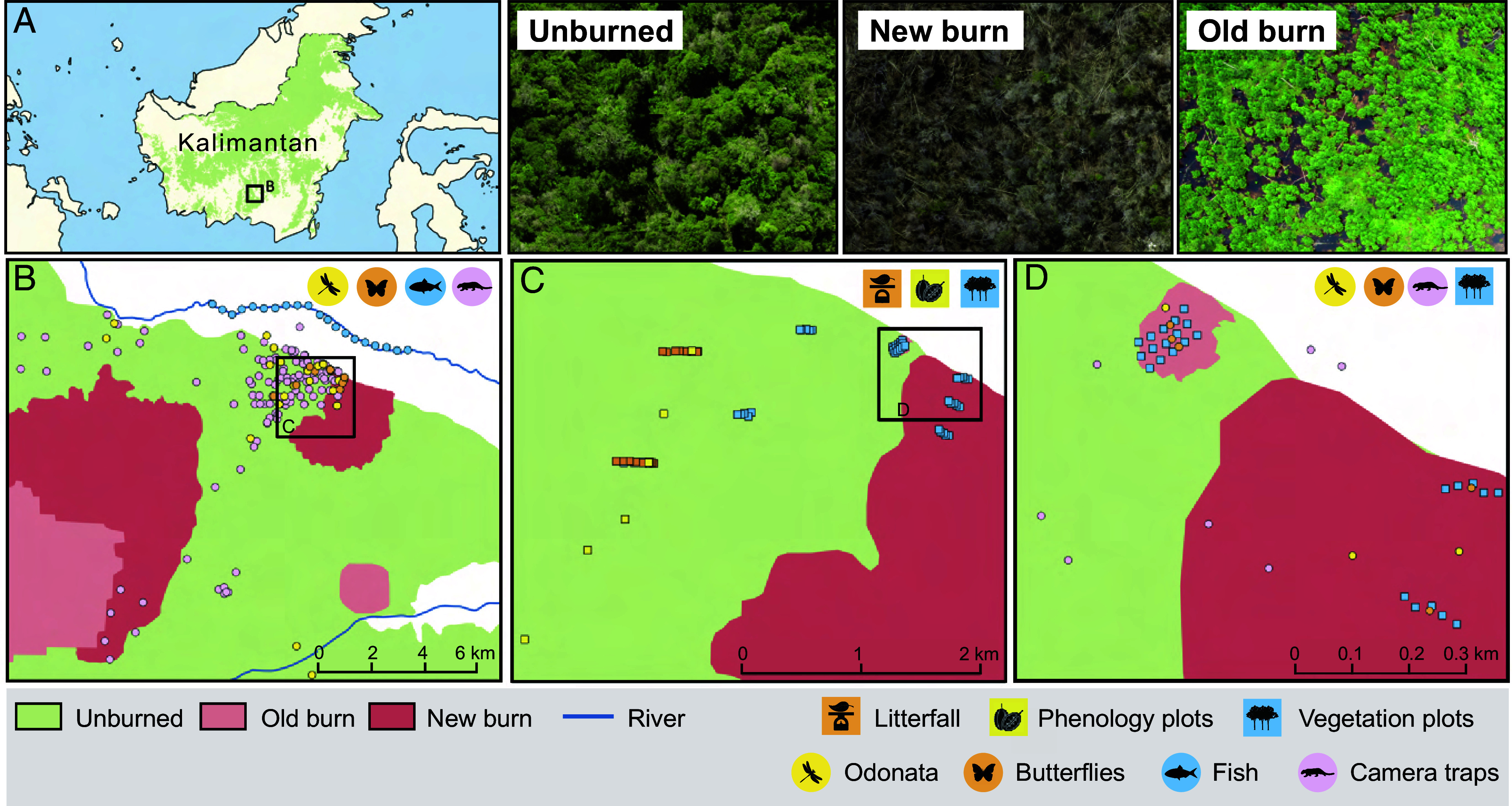
Map of the study site, illustrating survey locations within Sebangau National Park and on Borneo (*A*), and at relevant spatial scales (*B*–*D*). The study area is centered around 2°19′00″ S and 113°54′29″ E and comprises low-lying (5 to 25 masl), ombrogenous peat-swamp forest. Parts of the forest have burned intermittently, creating a mosaic of predominantly unburned forest interspersed with areas burned at different times. For surveys conducted using transects, points indicate the central location of the transect. Central butterfly survey locations indicated in (*B*) refer to indirect, time-series data collection locations, whereas those in (*D*) indicate locations for direct comparisons of burned and control conditions. Fish sampling was conducted along the Sebangau River [average water body width 30 m and depth 5.4 m around our survey locations; (32)]. See *SI Appendix*, Table S1 and *Appendix S2* for methodological and sample size details for all datasets. Map data sources: forest cover (33); burned areas extracted from dNBRI Landsat imagery (Landsat-8 OLI/TIRS image courtesy of the U.S. Geological Survey) and ref. 34; rivers and conservation areas courtesy of Indonesian Geospatial Agency (SIGAP KLHK).

Here, we conduct a comprehensive assessment of fire impacts on the structure, composition, functioning, and biodiversity of a forested tropical ecosystem. We have focused on a 16-y dataset from a 320-km^2^ tropical peat-swamp forest research area in Indonesian Borneo (Fig. 1). The area is particularly important to study because of its fire history, comprising burned areas in various stages of recovery interspersed within unburned forest subject to indirect impacts of fire within the wider landscape. Tropical peatland fires are exacerbated by peatland drainage and are typically anthropogenic in origin, in this region being predominantly driven by slash-and-burn agricultural practices, plus use of fire to clear areas for fishing and in land tenure conflicts (29–31). Peatland fires may smolder for days or even months, until extinguished by human intervention or rain.

Drawing on a matched analytical framework, we examine the ecological impacts of burn events and extend this to explore the fire regime characteristics driving ecological disruption and the potential for natural recovery in fire-sensitive forest ecosystems. We synthesized 27 ecological component datasets to explore how fire affects core ecosystem properties (i.e., the abiotic, structural, and functional alterations directly attributed to fire exposure) and biodiversity (i.e., an emergent feature of both fire and the alteration of the ecosystem properties, partitioned into forest specialist and all species) (*SI Appendix*, Table S1a). We systematically compared areas subjected to a recent burn event (new burn; burned 1.5 to 5 y prior to surveys), with those recovering from historical fires (old burn, burned 10 to 21 y prior) and an adjacent relatively undisturbed peat-swamp forest (unburned).

To examine the sensitivity of ecosystem dynamics within forest areas to temporal variations in fire within the wider landscape (fire frequency and intensity based on satellite data), we collated longitudinal data on a further nine ecological components, comprising 236 sampling locations spanning a 16-y period, thus allowing for detection of both immediate and graduated responses to indirect fire impacts (*SI Appendix*, Table S1b). Of particular interest was the impact of large-scale megafires, which we quantified using a combination of spatial (distance to most recent megafire) and temporal (time since last megafire) measures to understand the extent to which the indirect impacts emerging from megafires permeate into adjacent habitat. The forest sampling locations incorporated in this part of the study were positioned between 0.5 and 8.75 km from burned areas within the wider landscape (Fig. 1). Using dynamic statistical frameworks, modified to account for imperfect detection where appropriate, we reveal how tropical peat-swamp forests are affected by the spatial and temporal footprint of fire.

This integrated spatiotemporal analytical framework enables us to test the hypotheses that fire impacts in forested tropical ecosystems 1) cause deterioration to ecological components in burn-affected areas; 2) are mediated by fire regime characteristics, which indirectly extend the spatial footprint of fire into adjacent unburned habitat; and 3) demonstrate some evidence of recovery following longer postfire intervals. Our results provide detailed insights into the impacts of fire in tropical ecosystems and their potential for recovery, while demonstrating the importance of enhancing fire management efforts in an increasingly flammable world.

## Results

### Direct Impacts of Fire on the Peatland Ecosystem and Potential for Recovery.

Burned forest (both new and old burn treatments) was characterized by diminished ecosystem properties and biodiversity compared to unburned controls (*SI Appendix*, Fig. S1; see also *SI Appendix*, Figs. S2 and S3 for modeled mean values for all variables). We observed altered microclimatic conditions, a proliferation of nonforest vegetation, and an erosion of forest attributes and biodiversity, which disproportionately affected forest-specialist taxa. Comparable effect sizes between aggregate ecosystem property and biodiversity variables implied that impacts were consistent across both abiotic and biotic components of the forest (*SI Appendix*, Fig. S1). Moreover, at the aggregate level, there remained a high degree of overlap between new and old burn treatments in the effect sizes for changes in ecosystem properties and biodiversity (both all species and forest specialists), implying little evidence of postfire recovery (*SI Appendix*, Fig. S1).

**Fig. 2.**
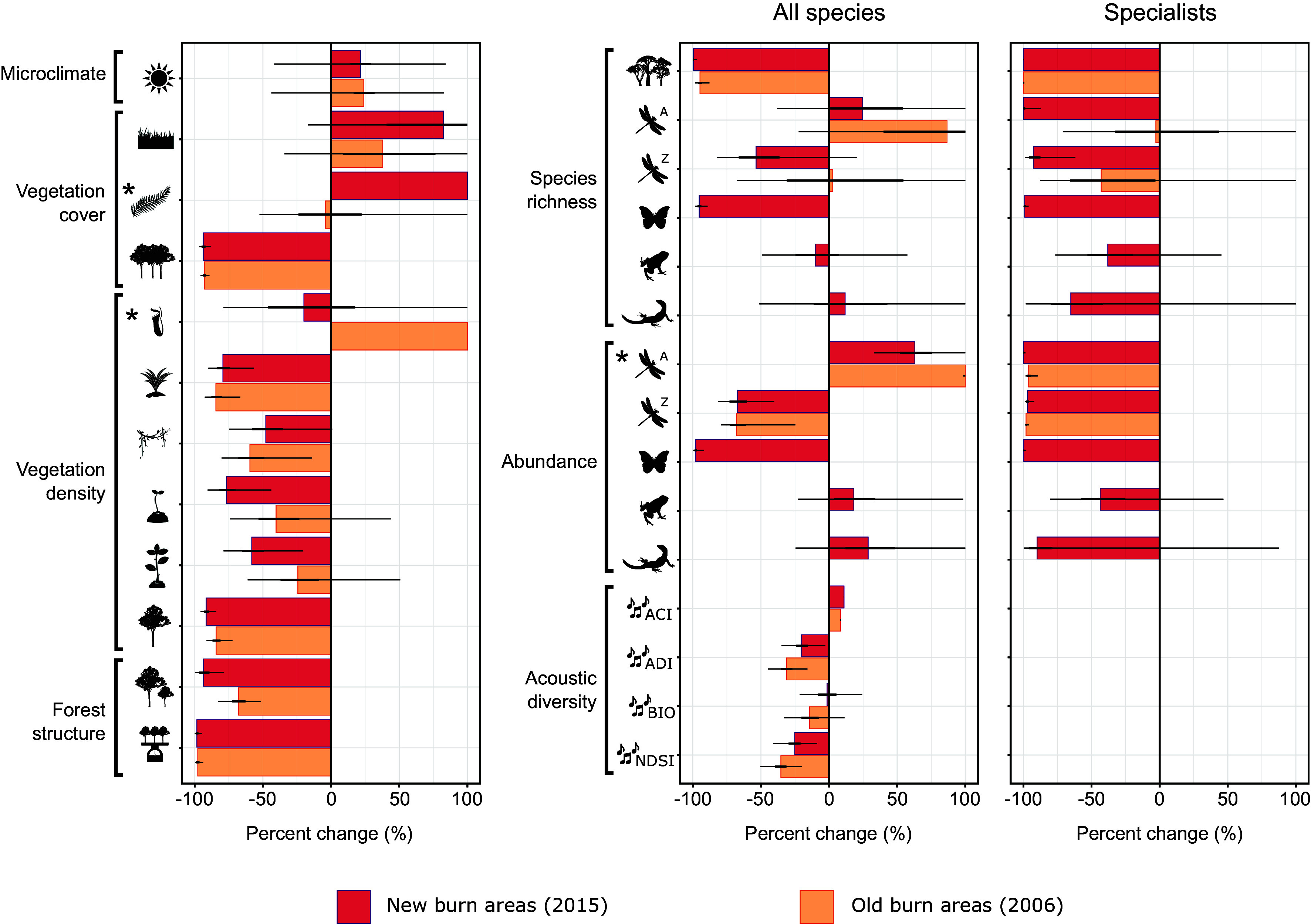
Percent change of ecosystem properties (*Top* to *Bottom*: maximum daily temperature, grass/fern/canopy cover, pitcher/pandan/liana/.seedling/sapling/tree density, canopy height, and aboveground biomass) and biodiversity characteristics (*Top* to *Bottom*: species richness and abundance of trees/dragonflies/damselflies/butterflies/amphibians/reptiles, Acoustic Complexity Index (ACI), Acoustic Diversity Index (ADI), Bioacoustic Index (BIO), and Normalized Difference Soundscape Index (NDSI)) in burn treatments relative to unburned forest controls (vertical dashed black line). Burn treatments captured “new burn” areas subjected to a recent fire event in 2015 (red hues) and “old burn” areas recovering from fire activity dating back to 2006 (orange hues). Uncertainty is expressed using 75% BCIs thick black horizontal lines) and 95% BCI (thin black horizontal lines). Asterisks denote ecological components that exceeded 100% increases in burn treatments relative to unburned forest controls and for which BCI lines are therefore not visible: fern cover: 1,156% increase in new burn treatment (95% BCI: 910 to 1,464%); pitcher plants: 954% increase in old burn treatment (95% BCI: 639 to 1,390%) dragonflies: 290% increase in abundance in old burn treatment (95% BCI: 98 to 606%).

Coarse-scale aggregate trends masked considerable variation in the extent of fire impacts within and between ecological datasets. For ecosystem properties, the strongest effects of burning were evident for forest structure, tree density (across all stages of the life cycle), and canopy cover, which declined by 58 to 98% when compared to unburned forest (Fig. 2). However, these impacts tended to be more severe in newly burned areas, indicating some postfire recovery over the 9-y interval between burn treatments. Further evidence of compositional recovery was observed, with higher densities of both seedlings and saplings in old compared to new burn areas. Similarly, the rapid proliferation of invasive ferns observed in newly burned areas (>1,000% increase in cover compared to controls) was greatly reduced in old burn areas (Fig. 2). In contrast, daily maximum temperature was higher in both burn treatments compared to unburned controls (old burn: 24% increase; new burn: 21% increase), likely reflecting the substantial reductions in canopy cover (>90%) consistent across both burn treatments (Fig. 2).

Burned areas generally contained fewer species, occurring at lower abundances; a finding broadly consistent among biodiversity datasets. Impacts were most pronounced for nonpioneer tree species, which were completely lost from all new burn areas, and forest specialist invertebrates, which experienced declines of up to 99.9% in species richness and equivalent reductions in abundance (Fig. 2). To a lesser extent, herpetofauna communities also contained fewer species (38 to 65%) and exhibited population declines of between 43 and 90% in fire-affected areas, with reptiles demonstrating a greater sensitivity to burn events. Forest soundscapes indicated variable responses to fire among the acoustic indices quantified, but declines in the prevalence and intricacy of some biotic signals in burn treatments were notable (up to 25% reduction; Fig. 2). Evidence of postfire recovery was more limited for biodiversity, but when comparisons between the old and new burn treatments were available, tree species richness remained suppressed. In contrast, Odonata species diversity rebounded (including 50 and 98% recovery of forest-specialist damselfly and dragonfly species, respectively), albeit at reduced abundances, while avian soundscapes regained a degree of acoustic complexity (Fig. 2).

### Indirect Impacts: Temporal Variation in Peatland Ecosystem Dynamics Relative to Megafire Events.

**Fig. 3.**
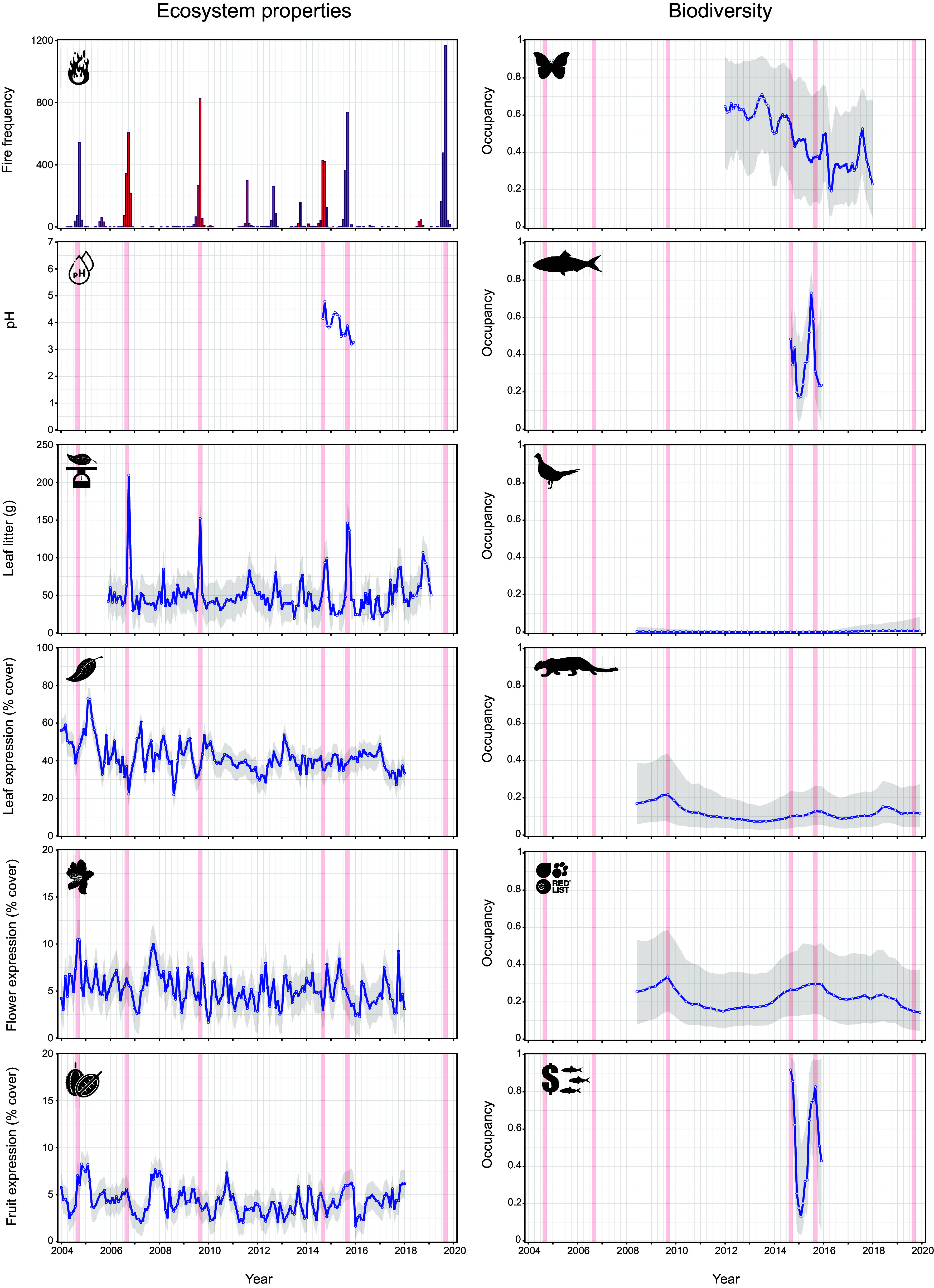
Temporal trends (blue lines) demonstrated by ecosystem properties (*Left* panel; *Top* to *Bottom*: fire frequency, river pH, leaf-fall, leaf/flower/fruit expression) and biodiversity (all species, *Right* panel; occupancy of butterflies, fish, ground-dwelling birds, medium-large terrestrial mammals, IUCN-threatened vertebrates, commercially valuable fish) in response to multiple megafire events (red vertical lines) across a 16-y time frame (January 2004 to January 2020). For biodiversity variables, occupancy reflects the probability that the species is present in the study landscape, where a value of zero indicates that the species is completely absent, and a value of one confirms that the species was present, during the observed time point. We present temporal summaries as posterior means of season-specific intercept terms (hollow blue points) and express uncertainty using 95% BCIs (gray ribbons). Trends for forest specialist species closely mirrored those of all species and are illustrated in *SI Appendix*, Fig. S4.

On average, 594 (range: 5 to 2,565) high-confidence fire detections were captured by MODIS satellites annually across the study site and a 25 km buffer surrounding it (total area: 625 km^2^). For Central Kalimantan province, this was extended to 11,779 (range: 528 to 38,002) annual detections. The ecological time-series datasets from forest areas were sensitive to the six megafire events occurring between 2004 and 2020 within this wider study landscape (all species: Fig. 3; forest specialists: *SI Appendix*, Fig. S4). We define megafires based on statistically anomalous peaks in fire regime characteristics, identified as months when both the frequency and summed radiative power of fire detections exceeded the 95th percentile of the historical fire profile (*SI Appendix*, *Appendix S1*). Comparing the average change in forest datasets pre- and post-megafire revealed that impacts on ecosystem properties were greatest at the 1-mo interval, where river pH became more acidic (posterior mean: −11.2%; 95% (Bayesian Credible Interval, BCI): −12.7 to −9.7%), and flower production (−21.3%, −59.5 to −2.8%) and leaf flush (−15.7%, −24.8 to −5.5%) declined, coinciding with an increase in leaf-fall (26.7%, 8.2 to 61.9%).

For biodiversity variables, we built temporal profiles of occurrence data, defined as the probability that a taxon, ecologically meaningful group (feeding guilds, threatened taxa, commercially valuable taxa) or species was present within the study area against the backdrop of historical megafire events. At the 1-mo interval, fish populations exhibited sharp decreases in occurrence (−67.9%, −84.5 to –39.7%), with noteworthy reductions in forest specialists (−73.5%, −83.0 to –59.4%) and commercially valuable species (−29.2%, −60.9 to −0.3%). A decline in butterflies during the same time frame was less severe (−14.0%, −35.8 to −0.02%), with forest specialist species generally more robust to megafires in the wider landscape (−5.7%, −27.3 to −21.8%). Mammals and ground-dwelling birds did not respond to fire events within the wider landscape when all species were considered together but were sensitive across longer timescales when analyses were based on threatened vertebrates (−19.8%, −40.3 to –0.07%, 6-mo interval) and forest specialist birds (−46.2%, −70.9 to −0.01%; 12-mo interval).

When analyses were restricted to the most severe megafire event in the time series (2009; Fig. 3), the above differences were exacerbated for most groups. Within 1 mo following the 2009 megafires, flower and leaf production reduced by 45.4% (−52.8 to −37.6%) and 39.5% (−43.2 to −35.9%), respectively, while across longer timescales, fruits became less prevalent (−21.4%, −32.9 to −6.6%; 9-mo interval). Declines of threatened vertebrate species escalated (−29.5%, −49.6 to −3.2%; 9 mo-interval), underpinned in part by a gradual erosion in mammal occurrence (−23.7%, −48.4 to −0.01%; 12-mo interval for all species, with no decline observed for forest specialists). However, general taxonomic trends often obscured idiosyncratic responses at the guild and species levels (*SI Appendix*, Figs. S6–S10). For example, over 12 mo following the 2009 megafires, Sunda clouded leopard (*Neofelis nebulosi*) populations declined dramatically (−47.8%, −67.2 to −19.4%; *SI Appendix*, Fig. S10), while herbivorous mammal occurrence increased by 69.5% (15.8 to 123.2%; *SI Appendix*, Fig. S6), demonstrating that some species have capacity to capitalize on the ecological opportunities presented by fires.

**Fig. 4.**
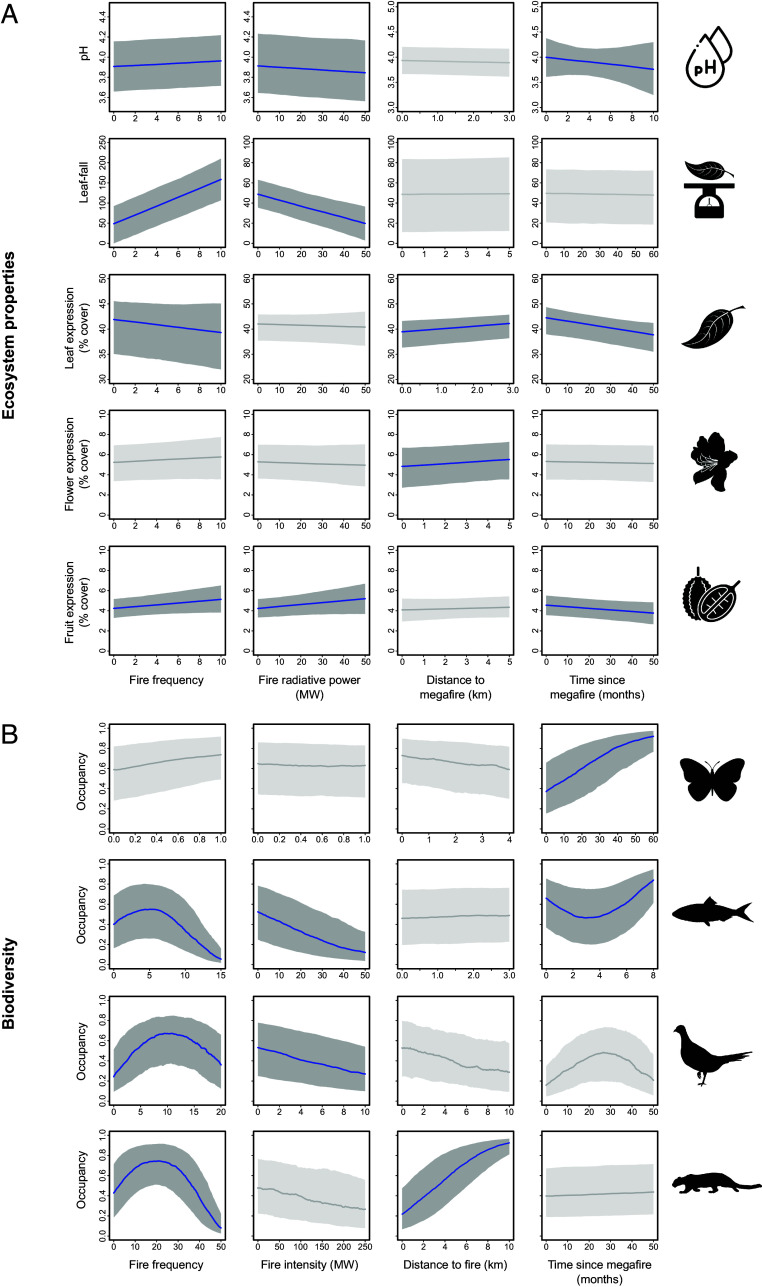
(*A*) Ecosystem property and (*B*) biodiversity responses to fire properties (fire frequency and fire radiative power) and the spatiotemporal proximity to extreme thermal events (distance from megafire and time since last megafire). Occupancy reflects the probability that the species is present in the study landscape, where a value of zero indicates that the species is completely absent, and a value of one confirms that the species was present, during the observed time point. Solid blue lines denote the mean of the posterior distribution while gray ribbons denote uncertainty, expressed using 95% BCIs. Noninfluential parameters are presented in light gray. For definition of symbols, see Fig. 3. Trends for forest specialist species are illustrated in *SI Appendix*, Fig. S5.

The sensitivity of forest ecological components to megafires within the wider landscape was driven by various, and often multiple, aspects of fire regimes, as illustrated in Fig. 4. For ecosystem properties, patterns of river pH, leaf-fall, and fruit production were most influenced by fire intensity (Fig. 4*A*). Fires characterized by a high radiative power resulted in acidic rivers, reduced leaf-fall, and greater fruit production. We also found moderate support for an influence of fire frequency on ecosystem properties (Fig. 4*A*), with rivers becoming less acidic, fruit production increasing, leaf flush decreasing, and leaf-fall increasing as fires became more prevalent. When we extended the analysis to include the distances of our forest survey locations from megafire-affected areas (*SI Appendix*, *Appendix S4.3.5*), we found that flowers and leaf flush tended to be more abundant when fires were more distant (Fig. 4*A*). With respect to time, fruit and leaf flush production diminished and rivers became more acidic as more time had elapsed since the last megafire event (Fig. 4*A*).

Biodiversity was highly sensitive to fire properties within the broader landscape, reflected in the occupancy responses of fish, ground-dwelling birds, and medium–large mammals (Fig. 4*B*). These taxa exhibited consistent nonlinear associations with fire frequency, indicating a degree of fire tolerance up to an inflection point, beyond which occupancy began to decline as fire frequency increased. Inspection of the inflection points relative to fire frequency indicates that more mobile species (e.g., mammals) tended to have a greater tolerance to fire than more sedentary taxa (e.g., ground-dwelling birds, Fig. 4*B*). Fish and birds were also sensitive to fire radiative power, becoming less prevalent with increasing fire intensity in the landscape. Butterfly occurrence was best modeled by spatiotemporal proximity measures (Fig. 4*B*), demonstrating higher occupancy with increasing time since megafires. The non-liear association between fish occupancy and time elapsed since the last megafire implies that fish populations were heavily impacted by megafires initially, but began to rebound around 4 mo after the event (Fig. 4*B*). Species-specific associations underpinning these coarse taxonomic responses are presented in *SI Appendix*, Figs. S8–S11. When only forest specialist species were considered, we found broadly consistent responses to fire incidence and properties across all taxonomic groups (*SI Appendix*, Figs. S4 and S5).

## Discussion

Ecosystem-scale syntheses of tropical forests have been rarely featured in fire impact assessments, despite these habitats being poorly adapted to, and heavily impacted by, fire (7, 8). We have compared newly burned, old burned, and unburned areas in a 320 km^2^ study area, and in so doing reveal the pervasive impacts of fire in tropical forest, involving the progressive deterioration of both ecosystem properties and biodiversity with important implications for recovery. For forest habitat in the vicinity of burned areas, we show that indirect ecological disruption is driven by both the frequency and intensity of the fire regime in the wider landscape, which often act together to erode the forest’s biological value and recovery potential.

### Ecological Impacts of Fire.

Our data indicate that the peat-swamp ecosystem experienced a cascading response to fire, with burned areas characterized by the loss of large standing trees, triggering a sequence of structural, microclimatic, and compositional alterations. Tropical trees generally lack specialized traits to withstand fire damage (35), resulting in substantial mortality (13), as evidenced by the complete loss of nonpioneer tree species from new burn areas in our study. Tree loss leads to architectural simplification in tropical forests, increasing light availability, and creating hotter, drier microclimatic conditions, favoring nonforest vegetation and pioneer species (11, 28). This includes invasive ferns, which rapidly colonize and dominate the postfire vegetation community in tropical peatlands, hindering native tree seedling establishment (15). High community turnover and disruption to plant demographic processes can result in long-term reductions in net primary productivity, nutrient cycling, and carbon storage in fire-affected forest areas (9, 36, 37). Similar postfire trajectories have been documented in Amazonia (e.g., refs. 14 and 16), indicating that this may be a generalized response in fire-sensitive forest formations.

Our results emphasize the sensitivity of tropical wildlife to fire, although the extent and magnitude of fire impacts varied across taxa and were difficult to generalize, in common with other assessments (8, 24). Across all taxonomic groups we assessed, forest specialists were found to be highly sensitive to the direct impacts of fire, suggesting that burned areas undergo compositional shifts in wildlife communities favoring disturbance-tolerant generalists. Such biotic homogenization is well documented in degraded habitats and has the capacity to exacerbate disturbance impacts if the remaining generalist species cannot provide compensatory ecological functions (38). The most pronounced impacts in our study were on aquatic fauna (fish), as rivers became more acidic from the leaching of dissolved organic carbon through burning (39). Fire impacts can be especially acute in freshwater systems as disruption to water quality and sediment flux propagates downstream (e.g., Australia: ref. 40), which may have particularly important impacts on local communities in tropical peatlands, given their often high reliance on fishing (41).

Wildlife responses to fire emerge from a suite of direct and indirect drivers that can act in isolation or synergistically. Fire can cause substantial direct mortality for tropical taxa (e.g., Brazil: (17)), many of which do not possess the response strategies to detect and escape from incipient burn events (18, 42). Fire can also affect wildlife indirectly through the disruption of forest phenological events and microhabitat conditions, compromising habitat quality, microclimatic suitability, and resource provisioning, which have been documented to have insidious effects on animal populations in the Amazon (43, 44). Moreover, exposure to toxic haze may be a pervasive, underappreciated threat affecting wildlife far beyond the burn extent, with reports indicating a capacity to impact animal behavior (19) and human health (45). Nevertheless, focusing on coarse trends in taxa belies complex species-specific responses. For example, fire in the wider landscape seemingly benefitted herbivorous mammals within our study time frame (*SI Appendix*, Fig. S6), presumably due to increased foraging opportunities, but these effects were reversed when all mammals were aggregated into a single taxonomic unit (Fig. 3).

Fire impacts are mediated by the discrepancy between historical and current fire regimes (6), amplifying concerns over the proliferation of fire activity in many tropical regions in recent decades (3, 25). This concern is mirrored on Bornean peatlands, where analysis of peat cores indicates that fire has been a rare phenomenon over most of the last 30,000 y, but has increased markedly in recent centuries alongside an increased human presence in the region (46, 47). Our time-series analyses demonstrate that increases in the frequency and intensity of burn events within the wider landscape are associated with the deterioration of plant phenological processes, water quality, and biodiversity in forest areas. Moreover, these ecological impacts were most pronounced in the aftermath of megafire events. Studies in the Amazon have shown that recurrent, high-intensity fires amplify the structural and compositional downgrading associated with burn events (16), exacerbating downstream effects on ecosystem processes and wildlife persistence (6, 11). For example, we found that indirect fire impacts were particularly acute for threatened vertebrates, contributing toward broader concerns that uncontrolled megafires may elevate the risk of species extinctions, even beyond the burn extent (48). While we observed ecosystem properties in forest areas to be able to recover quickly to predisturbance levels following megafires in the broader study landscape in Borneo, biodiversity in the Amazon has been shown to experience a gradual erosion following fire, with potentially long-term consequences for wildlife-mediated processes underpinning habitat recovery (20). Taken together, these results indicate that fire management should actively prioritize tropical peatland areas that frequently burn to minimize the risk of intense fires over time and prevent irreversible state shifts.

### Postfire Recovery.

Ecological recovery of tropical forest following fire is largely determined by the retention of large reproductive trees and seedling recruitment (49). Based on these criteria, our study provides a mixed prognosis for natural postfire recovery in tropical peatlands. On the one hand, the sustained absence of large trees is known to cause deficits in seed production for native species (49), manifesting in our case as a sustained decline in nonpioneer tree diversity across the 12-y regeneration time frame studied. On the other hand, our results provide some evidence of compositional recovery, with some ecosystem (e.g., canopy height) and biodiversity (e.g., damselflies) components at least partially recovering over relatively short time frames. Light-demanding pioneer vegetation also became less prevalent over time, resulting in increased seedling recruitment, with sustained growth indicated by concomitant increases in sapling density. Given that immature trees are extremely vulnerable to fire-related mortality, including in wetland forest areas such as the Pantanal (50), the extent to which this recovery can be maintained will be dictated by the capacity of each peatland to resist future fire.

The demonstrated links between habitat structure, microclimate and biodiversity limited the ability of vegetation and wildlife to rebound from fire within our 12-y study period, particularly for forest specialist species. However, it is reasonable to expect fuller recovery of biodiversity over decadal or centennial time frames. For example, we found higher seedling and sapling densities in old compared to new burned areas, which over longer time periods and in the absence of repeated fire, should lead to increased density of large fruiting trees, providing resources for frugivores to return. Indeed, in nearby areas on Borneo, recovery of tropical peatland tree diversity was possible two to three decades after fire, though even relatively infrequent repeated fire (50 to 100 y interval) may substantially suppress recovery (28). Paleoecological evidence indicates an ability for plant communities to persist following fire several thousand to several hundred years ago, while also revealing declines in peat-swamp forest and an apparent lack of regeneration associated with more recent increased anthropogenic influence and fire incidence (46, 47). Our data illustrate the sustained decline of nonpioneer tree species up to 12 y following fire, emphasizing that full recovery of species diversity following fire is likely to be a slow process. In turn, this reiterates the need for fire management to be considered an integral part of tropical peatland protection, restoration, and revegetation efforts (31).

### Managing Tropical Landscapes for Fire.

Despite the importance of appropriate management strategies to safeguard fire-sensitive ecosystems, efforts to suppress forest fires often have limited success (10). Furthermore, it is becoming increasingly recognized that positive ecological and social outcomes arise from integrated policies that prioritize fire prevention and habitat restoration concurrently (31, 51). In tropical regions, most fires are of anthropogenic origin (2), therefore policy mechanisms that limit fire use in agriculture and tackle deforestation, such as Indonesia’s 2011 moratorium on forest and peatland conversion, are fundamental. Policy can be strengthened further by augmenting preventative management with restoration actions to prevent recurring fire and arrested succession arising from feedback loops. We demonstrate that postfire recovery in tropical peatland is a gradual process and areas subjected to frequent/intense fires may not fully recover unassisted, at least across human-relevant time frames (22). For example, across Borneo, over 2.5 million hectares of peatland have been documented to persist in a fern-dominated state for nearly 20 y (52).

Restoring the water table of degraded peatlands is a critical first step to prevent future fires, though efforts to block drainage canals dug for agricultural conversion or timber extraction may in some cases lack community support (31, 32, 53). Further interventions may be required to remove biophysical barriers to succession and enhance vegetation diversity. For example, natural regrowth can be supplemented with cost-effective direct seeding of native species from adjacent unburned forest (54), and recent syntheses provide a valuable knowledge base for increasing the success of active tree planting to revegetate burned tropical peatland areas (55). Moreover, identifying and maintaining connected areas of unburned habitat (“fire refugia”) can provide a source of seeds, while also reintroducing vertebrate-mediated processes to fire-affected areas (56). We present a pathway to fire prevention and restoration in forested tropical habitats, with an emphasis on peatlands, but it is also important to acknowledge that fire management must be an adaptive process tailored to the socioecological context. A one-size-fits-all approach is therefore unlikely to be effective.

An important first step in such an adaptive process is to develop a detailed understanding of how fire impacts forest ecosystems, the specific aspects of the fire regime driving ecological disruption and the potential for natural recovery. Here, we find that forested tropical ecosystems are highly vulnerable to recurrent, high-intensity fires and demonstrate that fire-affected ecosystems are capable of natural recovery, but assert that management actions may be required to break fire feedback loops and prevent arrested succession. Capitalizing on lessons learned here and elsewhere in the tropics (6, 24), and interpreting these across a range of socioecological contexts, will be critical in reducing the prevalence of uncontrolled forest fires and mitigating their impacts across the tropical realm.

## Materials and Methods

### Study Site.

Field data were collected in the Natural Laboratory of Peat-Swamp Forest (NLPSF) special research zone within the Sebangau National Park, Central Kalimantan, Indonesia (Fig. 1). The data collection area comprises ombrogenous mixed peat-swamp forest with peat depths ranging from 0.4 to 2.6 m (57) and experienced 40 y of logging prior to formal protection in 2004 (58). Timber-extraction canals (typically 1 to 2 m wide and 0.3 to 1.3 m deep) remain, however, causing continued peat drainage and heightened fire risk (32). Despite high annual rainfall (~3,000 mm) and ongoing hydrological restoration efforts, parts of the forest have therefore burned intermittently, creating a mosaic of predominantly unburned forest interspersed with areas burned at different times. The site is bordered in the north by the blackwater Sebangau River, which originates in the swamp and runs for ~150 km, before discharging into the Java Sea (38). No human settlements are present within the field data collection area, though the village of Kereng Bangkirai (population ~5,500) is situated ~2.5 km northwest.

### Characterizing Spatiotemporal Fire Regimes.

We developed a historical profile of fire regimes across Central Kalimantan between November 2000 and January 2020 using fire detection data obtained from the Moderate Resolution Imaging Spectroradiometer (MODIS) thermal anomalies MCD14ML product Collection 6. These data correspond well with ground-truthed burned areas in tropical peatlands (59). Detection data comprised the date, time, location, and frequency of active fires at 1 km spatial resolution, plus ancillary information on fire intensity (radiative power) and detection confidence. To avoid false detections resulting from nonfire heat signatures, we excluded low confidence thermal anomalies [<30%; (25)]. The resulting dataset comprised 235,600 fire detections across the 20-y period, with 90% of observations concentrated within a distinct fire season corresponding to late dry season (August-October), which may be extended by a month or more during drought-affected years associated with El Niño events. We defined megafire periods as months with statistically anomalous fire activity at both provincial (Central Kalimantan: 153,564 km^2^; human population 2.4 million) and local scales (NLPSF boundary augmented with a 25 km buffer; total area: 625 km^2^_;_ human population ~146,000). This resulted in six megafire events, which matched well with other reports of major fire events in the region (23). Full details of this procedure are provided in *SI Appendix*, *Appendix S1*.

### Field Data Collection.

To assess direct fire impacts, we compiled fire treatment datasets for 27 ecological components across 181 sampling locations between April 2017 and September 2021 (*SI Appendix*, Table S1), representing areas affected by a recent burn event in 2015 (“new burn”; *N* = 72) and those recovering from fires up to and including 2006 (“old burn”; *N* = 27). Burn treatments therefore captured immediate fire impacts and the potential for recovery in burned areas. For comparison, baseline data were also collected from forest areas with no history of fire (“unburned”; *N* = 82). Datasets were not all collected across all sampling locations, or at the same time, producing variation in sample sizes and postfire intervals between treatments.

To explore temporal variation in peatland ecosystem dynamics relative to fire regimes within the landscape, we compiled ecological time-series datasets spanning a 16-y period (September 2003 to December 2019; *SI Appendix*, Table S1 and *Appendix S2*). This time frame captures variations in annual fire regimes typical of the region, including multiple megafire events. Time-series datasets encompassed 578 temporally replicated surveys across 236 sampling locations for nine ecological components (*SI Appendix*, Table S1). All datasets coincided with at least two megafire events but sampled different locations and time frames within the 16-y temporal window. Full methodological details for all fire treatment and time-series datasets listed in *SI Appendix*, Table S1 are provided in *SI Appendix*, *Appendix S2*.

### Modeling Framework.

#### Direct impacts of fire and the potential for postfire recovery.

Fire treatment data for ecological components were summarized as mean values across sampling locations. In contrast, temperature data were calculated as maximum daily values, to capture microclimatic extremes that may compromise environmental suitability for vegetation and wildlife. Biodiversity characteristics were expressed as species counts and abundance estimates, averaged across temporal replicates where applicable, to evaluate compositional and population-level variability across treatments. Species richness estimates were bias-corrected for uneven survey effort using sample-based extrapolation [*SI Appendix*, *Appendix S4.1.1*; (60)].

We developed pairwise comparisons of ecological components in burn treatments relative to unburned forest controls, using standardized mean differences (Hedges *g*), modified to account for heteroscedasticity between treatments (*SI Appendix*, *Appendix S4.1.2*). This measure accounted for variation in sample sizes and reconciled different measurement units between datasets. To account for nonindependence among effect sizes for datasets with multiple treatment groups (i.e., containing both new and old burn conditions), sample sizes in burned forest were adjusted by dividing them by the number of times controls were compared with burn treatments (61). For consistency among datasets, negative effect sizes represented detrimental impacts of fire on peatland ecosystems, while positive effect sizes conveyed ecological benefits of fire (*SI Appendix*, *Appendix S4.1.2*).

To examine the relative consequences of fires on peatland ecosystems, we summarized effect sizes using a hierarchical mixed effects meta-analysis. This framework controlled for higher precision in datasets containing a greater number of statistical replicates. We specified random effects for data type (i.e., ecosystem property or biodiversity), to understand coarse-scale ecological responses to fire; ecological components, to assess fire impacts on specific aspects of peatland ecosystems; and individual observations, to explicitly model residual variance. We quantified overall fire impacts on peatland ecosystems by weighting dataset-specific effect sizes by the inverse of their variance, plus the interdataset variance (62).

To quantify proportional changes in each ecological component, we constructed generalized linear mixed-effects models (GLMMs). GLMMs were selected because they extend traditional linear regression frameworks to accommodate data types common to ecological assessments (counts, proportions). All models were specified as mean parameterizations describing each component as a linear function of fire treatments. We also included a spatial random effect to account for clustered sampling where necessary. Proportional changes between unburned controls and burn treatments were calculated post hoc using the formula: ((α_burned_ − α_unburned_)/α_unburned_) × 100), where α represents model-estimated treatment means. See *SI Appendix*, *Appendix S4.2* for further details on GLMM specification.

#### Indirect fire impacts: Temporal variation in ecosystem properties and biodiversity relative to megafire regimes.

Prior to modeling, time-series datasets were partitioned into discrete primary sampling occasions, termed sampling seasons, based on a combination of sampling frequency, life history characteristics, and meaningful periods of fire activity (3 mo for terrestrial vertebrates; 1-mo for all other ecological components). Across all datasets, seasons operated consecutively to provide complete temporal coverage across the data collection period. For biodiversity datasets, we constructed species-specific detection histories for each taxonomic group by pooling detection/nondetection data into discrete sampling occasions nested within seasons. Full details of time-series data processing are provided in *SI Appendix*, *Appendix S4.3*.

To build a temporal profile of fire regimes for each time-series dataset, we extracted seasonal summaries of fire frequency and intensity (fire radiative power: mean, max, SD, sum) from fire detection data. Due to high levels of collinearity between intensity metrics (|*r*| > 0.7; VIF > 5), we selected the sum of fire radiative power to represent this aspect of the fire regime, as it consistently outperformed competing measures during bivariate exploratory analysis (*SI Appendix*, Table S2). To estimate the extent to which megafire impacts radiate across space and time, we calculated the time since last megafire (months) and Euclidean distance (km) from all fire detections occurring within the previous megafire period for each sampling location. Throughout, we extract covariates across buffer radii selected using scale optimization methods (*SI Appendix*, Table S2). This approach addressed considerable uncertainty regarding the appropriate scale of effect for fire impacts (63), which has been shown to extend in excess of 3 km for certain taxa (64). All fire covariates were centered around their mean values and scaled to one-unit standard deviation to place them on a comparable scale and improve computational efficiency.

We implemented GLMMs to quantify temporal trends in ecosystem properties against the backdrop of megafire events. GLMMs were selected due to their capacity to incorporate random effect structures that compensate for nonindependence in temporal assessments arising from repeat observations at the same sampling location. Annual trends in ecosystem properties were estimated using season-specific random intercepts. We also incorporated a spatial random effect to account for spatially structured variation due to unobserved ecological factors.

To examine biodiversity responses, we employed hierarchical multispecies occupancy models to provide inference at multiple taxonomic levels, improve estimation precision for infrequently observed species and explicitly account for imperfect detection (65). We described the occurrence state (the true presence/absence of a species) on the logit scale using season-specific intercepts and spatial random effects terms. We introduced a random walk prior to the occupancy intercepts to improve estimation precision by allowing the sharing of information between consecutive seasons (following ref. 66). We described the detection process on the logit scale, using a season-specific intercept and a measure of survey effort to address differences in sampling intensity between seasons. We summarized temporal trends by taxon, IUCN threat status (ground-dwelling birds/mammals), ecologically meaningful groups (mammals: feeding guilds), commercial value (fish only, based on data from refs. 32 and 41), and species. Details of group assignment are presented in *SI Appendix*, Table S3.

Building on this model structure, we constructed a further two candidate models for each time-series dataset to understand the specific aspects of fire regimes driving temporal trends: 1) a fire properties model, to capture the additive effects of fire frequency and intensity; and 2) a spatiotemporal proximity model, to understand how distance in space and time mediates megafire impacts. Across all models, we incorporated quadratic terms where appropriate to model nonlinear associations. To provide quantitative comparisons between candidate models, we calculated Watanabe-Akaike Information Criterion (WAIC), which is a within-sample model selection criterion, robust to statistical frameworks containing latent parameters. Throughout, we considered models with ΔWAIC < 2 to have comparable statistical support and 2 < ΔWAIC < 8 to have moderate support (*SI Appendix*, Table S4 and *Appendix S4*).

All analyses conducted were specified within a Bayesian framework, implemented in rstan (hierarchical mixed-effects meta-analysis) and JAGS (all GLMMs and occupancy models) called through R version 4.0.2. Model development, structure, specification, and evaluation details are presented in *SI Appendix*, *Appendix S4*.

## Supplementary Material

Appendix 01 (PDF)

## Data Availability

Relevant data files have been deposited in the Environmental Information Data Centre (67).
