# Supplementary material for: Impacts of fire and prospects for recovery in a tropical peat forest ecosystem
Source: Proc Natl Acad Sci U S A. 2024 Apr 15;121(17):e2307216121. doi: 10.1073/pnas.2307216121 (PMC11047076; doi:10.1073/pnas.2307216121)
Supplement: Supplementary file 1 — Appendix 01 (PDF) [file pnas.2307216121.sapp.pdf]

PNAS

Supplementary Information for

Impacts of fire and prospects for recovery in a tropical peat forest ecosystem

Mark E. Harrison[1,2], Nicolas J. Deere[1,2], Muhammad Ali Imron, Darmae Nasir, Adul, Hastin Ambar Asti, Joana Aragay Soler, Nicholas C. Boyd, Susan M. Cheyne, Sarah A. Collins, Laura J. D'Arcy, Wendy M. Erb, Hannah Green, William Healy, Hendri, Brendan Holly, Peter R. Houlihan, Simon J. Husson, Iwan, Karen A. Jeffers, Ici P. Kulu, Kitso Kusin, Nicholas C. Marchant, Helen C. Morrogh-Bernard, Susan E. Page, Ari Purwanto, Bernat Ripoll Capilla, Oscar Rodriguez de Rivera Ortega, Santiano, Katie L. Spencer, Jito Sugardjito, Jatna Supriatna, Sara A. Thornton, F. J. Frank van Veen, Yulintine, Matthew J. Struebig

[1] M.E.H. and N.J.D. contributed equally to this work.

[2] To whom correspondence may be addressed. Email: M.E.Harrison@exeter.ac.uk (M.E.H.); N.J.Deere@kent.ac.uk (N.J.D)

This PDF file includes:

Appendix S1: Defining a megafire

Appendix S2: Detailed field survey methods

Appendix S3: Classification of forest specialist species

Appendix S4: Statistical method details

Tables S1 – S5

Figures S1 – S12

References Cited in Supplementary Information

Appendix S1: Defining a megafire

We defined megafire periods as months demonstrating statistically anomalous fire activity based on fire detection data (1). To do this, we constructed statistical distributions across the historical fire profile based on monthly-aggregated values for fire frequency and radiative power. We then identified a megafire event as any month where the frequency and summed radiative power of fire detections exceeded the 95th percentile of these distributions at both provincial (Central Kalimantan) and local-scales (boundary of the Natural Laboratory of Peat-Swamp Forest special research zone: 625 km^2, augmented with a 25 km buffer). Megafires occurring across consecutive months were treated as a single event. As megafires are typically characterized by size, only megafire periods that were consistently identified across the above two scales were selected to ensure that statistically anomalous peaks in fire activity corresponded to large-scale burn events. This resulted in six megafire events (October 2004; October 2006; October 2009, September-October 2014; September-October 2015; August-September 2019), which matched well with other reports of major fire events in the region (2, 3).

Appendix S2: Detailed field survey methods

Here, we provide detailed descriptions of the methods underpinning the collection and compilation of ecosystem property and biodiversity data collected. We present data descriptions according to the analysis to which they contributed. Throughout, species identification protocols for biodiversity data from different taxonomic groups follow those described in (4).

Direct impacts of fire and the potential for post-fire recovery

The following data were collected from each of the three (or in some cases two) different fire treatments described in the main manuscript: new burn, old burn and unburned forest.

Ecosystem property data:

Microclimate (temperature)

Temperature was recorded from each treatment using DS1921G-F5 Thermochrons and a TDHC 400 Thermochron Reader from August to September 2018. Thermochrons were attached to the underside of passive acoustic monitoring units (see below) to avoid direct sunlight exposure, and enclosed in a silicon case. Temperatures recorded from inside cases did not differ from those recorded outside cases in pre-deployment tests. Temperature was recorded automatically every 30 minutes between sunrise and sunset, resulting in a total 168 records per treatment. We express variation in microclimate between treatments as maximum daily temperature (ºC) for each sampling day.

Vegetation

Vegetation composition, density and structure were estimated across 45 nested 10 x 10 m forest inventory plots (new burn = 15; old burn = 15; unburned = 15), established between February and May 2019. Within each treatment, three parallel transects were established and separated by a minimum of 100 m. Along each transect, five plots spaced 50 m apart were surveyed. The following vegetation characteristics were recorded for each plot:

1. Vegetation cover (proportion): canopy cover derived from visually estimated vertical strata of 15 m or greater was estimated at the plot centre by experienced observers using a densitometer. The

proportion of the terrestrial plot surface area occupied by grass and ferns was also estimated visually. All vegetation cover covariates were derived from the 10 x 10 m plot area.

2. Vegetation density (count m^{2-1}): across the nested plot structure, we counted the number of pitcher plants (*Nepenthes* spp.), large Pandanaceae plants, lianas (all previous within 5 x 5 m sub-plots), seedlings (height < 1 m; 2 x 2 m sub-plots), saplings (< 10 cm diameter at breast height, DBH, and height ≥ 1 m; 5 x 5 m sub-plots) and trees (≥ 10 cm DBH; 10 x 10 m plot). We only included living vegetation throughout these assessments. For comparative purposes, raw counts were standardised to density estimates by dividing vegetation counts by the respective plot areas.
3. Forest structure: we used tree height (m) and aboveground live biomass (t ha^{-1}) to characterise plot-level structural integrity. For each live tree encountered within the 10 x 10 m plots (*N*=200), tree height was estimated visually and assigned to 5-m height bands. We calculated biomass based on individual measurements of DBH (cm), tree height (m, representing the mid-point from bands estimated by experienced observers: 0-5, 6-10, 11-15 m, etc.) and wood-specific gravity (g $cm^{3\ -1}$; based on averages of species identity or most highly resolved taxonomic unit; (5)) using an established pantropical algorithm (6). Structural metrics were aggregated to the plot level as either average (tree height) or mean (aboveground biomass) values from individual-based measurements.

Biodiversity data:

Trees

We recorded the taxonomic identity of all live trees encountered (*N*=200) across the forty-five 10 x 10 m vegetation plots described above. This resulted in the identification of 45 tree species, distributed across 33 genera and representing 23 families. Of these, 15% of specimens (*N*=30) could not be resolved to species-level and so were discarded from further assessment.

Odonata

Adult dragonflies and damselflies were sampled across 22 transects measuring 250 m (new burn = 5; old burn = 2; unburned = 15) between January and July 2019. All transects were not surveyed in all months, which resulted in differences in survey effort between transect and treatments. Transects were walked at a slow pace, using butterfly sweep nets to capture and then release all individuals

encountered (7, 8). Following standard protocols for Odonata surveys (9), we avoided recaptures of the same individual by marking a wing of captured individuals with a permanent marker, adopting different coloured markers to differentiate between sampling periods (months). Individuals marked as captured in a previous month were only seen on three occasions and were excluded from the dataset. As the Anisoptera and Zygoptera sub-orders may show different responses to changes in habitat condition (10, 11), these were treated as separate taxonomic units in our assessment. Respectively for Anisoptera and Zygoptera, sampling resulted in 3,210 and 3,870 independent captures, represented by 22 and 25 species.

Lepidoptera: Papilionoidea

Butterflies were sampled along six transects (new burn = 3; unburned = 3) using fruit-baited traps set at 50 m intervals (12-14) between August 2018 and 2019. At each sampling point, traps were deployed in pairs and vertically stratified to simultaneously sample ground (1-2 m) and canopy (7-15 m) levels. During each survey month, traps were inspected for five consecutive days following deployment. Captured butterflies were marked and released, and recaptures of the same individual in the same month were excluded. Overall, 643 unique individuals were captured, comprising 16 species.

Herpetofauna

Amphibians and reptiles were sampled along six 1-km transects (new burn = 3; unburned = 3) from August to September 2021. Sampling along these transects comprised a combination of both diurnal (0800-1100h) and nocturnal (1900-2200h) surveys conducted for three consecutive days. Transects were partitioned into 250 m intervals, across which all visual and acoustic herpetofauna encounters were recorded. Throughout, we treat amphibians and reptiles as distinct taxonomic units. Sampling resulted in a total 685 herpetofauna encounters (amphibians= 427; reptiles = 258), represented by six and 12 species of amphibian and reptile, respectively.

Avian-focused soundscape indices from acoustic recorders

We used Song Meter SM4 (Wildlife Acoustics Inc.) autonomous recording units to record soundscapes in nine locations (new burn = 3; old burn = 3; unburned =3) between September 2018 and August 2019. Deployment schedules followed a rotational procedure whereby units were deployed for seven

consecutive days at one sampling location, following which they were repositioned to another sampling location within the treatment. This protocol enabled the simultaneous pairing of locations across the treatments. Because we were primarily interested in the avian component of the soundscape, recorders were set to activate during the hours of peak bird acoustic activity, with recordings thus obtained from 15 mins before sunrise for a duration of 2 hours, sampled at a rate of 24 kHz. We applied a 6 kHz maximum frequency limit to our data, to restrict our analysis to the typical reported avian frequency range (15), while omitting loud stridulating insects that frequently produced dominating sound bands above 6 kHz, which heavily skew acoustic indices (16). Each two-hour recording was split into 12 files of 10-min duration using Kaleidoscope 5 software. We calculated acoustic indices for each 10-min file, from which we derived a mean daily value for each index. Sound file spectrograms were scanned visually to identify potential disruptions that dominated the sound profile and may skew the acoustic indices derived, such as heavy rain, high winds, aircraft noise, people talking, loud cicada noise, static, and excessive and close animal movement (rustling). Where such potential disruptions were identified, the file was double-checked through playback, with disrupted files subsequently excluded from the analyses. We used the R package Soundecology (17) to derive four acoustic indices that are commonly applied to forest bird communities due to strong correspondence with manual bird surveys (18-20) and demonstrated sensitivity to fire-related disturbance (21, 22): Bioacoustic Index, Acoustic Complexity Index, Acoustic Diversity Index and Normalised Difference Soundscape Index.

Indirect fire impacts: Temporal variation in ecosystem properties and biodiversity relative to megafire regimes

Ecosystem properties:

River pH

Data on river pH was collected from 20 sample locations, spaced at 400 m intervals along a 7.6 km transect on the Sebangau River between September 2014 and December 2015 (temporal window: 16 months). No data were collected in October 2015 owing to local fire disruption. Variation in river pH was measured monthly using Hanna HI-98127 stick meters. Full details of the experimental design and sampling protocols are provided in (23).

Forest productivity (leaf-fall)

Litter-fall was collected from 16 x 1 m^2 traps separated by intervals of 50 m, along two unburned forest transects between December 2005 and January 2018 (temporal window: 159 months). During each monthly sampling event, litter-fall was separated into its constituent components (leaves, reproductive parts, branches < 5 cm diameter, bark, miscellaneous debris) and dried to a constant weight in a kerosene oven at ~40 C to obtain field dry weights (g). Throughout, we focus on leaf-fall, as this constituted the largest component of total monthly litter-fall and has a demonstrated sensitivity to fire impacts (24, 25). Data from December 2007 were discarded owing to disturbance of traps by local forest users. Full methodological details are provided in (26, 27).

Tree Leaf Flush and Reproductive Phenology

Tree leaf flush and reproductive phenology data were collected monthly from six permanent, unburned 0.4 ha forest inventory plots between September 2003 and January 2018 (temporal window: 173 months). No data were collected in October 2015 owing to local fire disruption. Surveys started around the 16th of each month. Data were collected from ground level by experienced observers using binoculars, who recorded the presence/abundance of fruit, flowers and leaf flush on each stem within the plots. Phenology scores represent the percentage of all stems surveyed in plots with fruit, flowers and new leaves. Fruit and flower production was enumerated across all trees ≥ 10 cm DBH, plus all figs and lianas ≥ 3 cm DBH rooted inside the plot (total plot area 2.4 ha). Leaf flush was recorded from all stems ≥ 6 cm DBH, plus all figs and lianas ≥ 3 cm DBH rooted inside the plot (total plot area 0.9 ha, representing a sub-section of the larger plot area used for surveying larger trees). No surveys were conducted in the peak fire months of October 2015 and September 2019 due to health and safety concerns, and low visibility preventing observers from accurately recording phenology data. Full details of sampling procedures are provided in (28, 29).

Biodiversity:

Butterflies

Butterflies were sampled monthly using 20 fruit-baited canopy traps positioned at 50-m intervals along two unburned forest transects between January 2012 and January 2018 (13). Monitoring protocols followed those described previously for butterflies in the "*Direct impacts of fire on peatland ecosystems*

and the potential for post-fire recovery" section of this Appendix. Sampling was suspended in January-February 2013 for logistical reasons and in September 2015 due to local fire disruption. Across 6,940 trap surveys, a total 5,368 butterflies were captured and released, representing 28 species.

Fish

Data on fish populations were collected between September 2014 and December 2015 (temporal window: 16 months), using the same survey design described above for river pH. Local fire disruption resulted in no data collection in October 2015. Following approaches adopted by local communities, fish were surveyed using 20 rectangular wire-mesh traps deployed on alternating banks of the river for five consecutive days. Traps were baited with a mixture of fermented soya bean and fermented shrimp paste. Captured fish were identified to species level, enumerated and released at the point of acquisition. Across 1,300 river surveys, a total 55,147 fish were captured, comprising 38 species. Full details of the experimental design and sampling protocols are provided in (23).

Terrestrial vertebrates

To monitor terrestrial vertebrates (ground-dwelling birds, i.e. species that typically forage at ground-level; and medium-large mammals, > ~1 kg) remotely-operated digital cameras (Cuddleback Expert and Cuddleback Capture IR) were deployed across 174 sampling locations between May 2008 and January 2020 (temporal window: 141 months). Camera traps were typically deployed using a gridded system, separated by an average inter-trap distance of 1 km. Deployments comprised a mix of paired stations (two parallel camera traps, placed 7-10 m apart) and single units, with passive infrared sensors consistently positioned at a height of 50 cm above the ground. Given that individual identification of species was not a primary objective of this assessment, paired stations were collapsed to represent a single sampling location. Cameras were placed on low resistance travel routes (established trails, man-made boardwalks) or key habitat features (water bodies, fallen logs) to maximise photographic captures (30). On average, units were deployed for 274 consecutive camera trap nights (CTN), though sampling effort was highly variable between locations (range: 4 - 3,609 CTN), resulting in a total survey effort of 47,689 CTN. Throughout the sampling period we identified 40 terrestrial vertebrates, comprising 13 ground-dwelling bird and 27 terrestrial mammal species.

Appendix S3: Classification of forest specialist species

Trees

Relatively few tropical peatland tree species are able to naturally recolonise and survive in burned peatland areas, with lists of these “pioneer” tree species provided in Table 7 in (31) and Table 1 in (32). Tree species not included in these references were thus considered to be non-pioneer species characteristic of tropical peat-swamp forest.

Odonata

Species listed under “Forest” in Appendix 1 of (33) were considered to be forest specialists. For species not listed therein, classification was based on information on habitat preferences provided in Appendix II of (7) or by (34).

Lepidoptera: Papilionoide

Species were classified as forest specialists where indicated as being captured solely in or strongly favouring forest sites in Appendix I of (7) and/or during pre-fire surveys in Appendix 1 of (35).

Herpetofauna

Classifications were based on descriptions provided in the *Habitat Type* section of IUCN Red List species accounts (accessed 20th September 2023). Species were considered to be forest specailists if only forest habitat was mentioned within this section.

Fish

We based our classifications of forest specialist fish on whether species had been captured exclusively in traps located in forest pools/canals by (23) in our Sebangau National Park study site. Data from these traps were not included within our main analyses, and were therefore considered sufficiently independent to support species classification. These preliminary designations were cross-referenced with habitat preferences derived from the IUCN Red List (accessed 20th September 2023). Here, species were considered forest specialists if records indicated a preference for “blackwater habitats associated with peat swamp forests”, “very soft, highly acidic (pH ~3-4) … heavily stained with tannins”,

submerged riparian vegetation, or other aspects of peat-swamp forest structure and a sensitivity to habitat disturbance.

Terrestrial vertebrates

For terrestrial vertebrates (medium-large terrestrial mammals and ground-dwelling birds) we defined ecological specialists based on forest-dependence and sensitivity to habitat modification according to evidence derived from (36), plus (37) for mammals.

Appendix S4: Statistical method details

S4.1: Meta-analysis

S4.1.1: Standardising species richness estimates

Given that empirical estimates of observed species are known to accumulate with increasing survey effort, we extrapolated species richness for sites with incomplete sampling using sample-size based rarefaction curves implemented in the R package "iNext" (38). This technique was preferred to rarefaction to prevent unnecessary disposal of data. Throughout, all extrapolations fell within criteria for reliable estimation (double the reference survey effort; (39)).

S4.1.2: Calculating and coding effect sizes

For quantitative comparison of ecological components between fire treatments, we calculated standardized mean differences (Hedges *g*: (40)), modified to account for heteroscedasticity between unburned forest controls and burn treatments (41).

$$g = J \times \left(\frac{\alpha_{unburned} - \alpha_{burned}}{SD_{pooled}}\right)$$

$$SD_{pooled} = \sqrt{\frac{(n_{unburned} - 1)SD^2_{unburned} + (n_{burned} - 1)SD^2_{burned}}{n_{unburned} + n_{burned} - 2}}$$

$$J = 1 - \frac{3}{4(n_{unburned} + n_{burned} - 2) - 1}$$

where *α, SD,* and *n* denote group means, standard deviation and number of sampling locations respectively for unburned controls and fire-affected habitats, SD_{pooled} is the aggregated standard deviation, applied to standardise mean differences in biodiversity value, and *J* is a bias correction factor.

For consistency between ecological components, all standardised mean differences were coded to reflect the underlying expectation that fire has a negative impact on peatland ecosystems. Accordingly, we inverted the sign of four ecological components where observed increases between forest and burn treatments reflected perceived negative impacts of fire:

- Maximum daily temperature: reduced canopy cover following fire is associated with increased range in, and maximum temperatures (and humidity) in forest ecosystems, which is known to negatively impact heat-sensitive species (e.g., butterflies: (42); anurans: (43)) and is expected to be detrimental for the large majority of forest-dependent species adapted to a more shaded, cooler environment.
- Grass and fern cover: high coverage of ferns and sedges is characteristic secondary vegetation in burned and heavily disturbed tropical peatlands that may inhibit woody plant regrowth (44, 45), and is not expected to provide suitable habitat or food resources for the majority of forest-dependent animal species.
- Pitcher plants: the carnivorous habits of *Nepenthes* species enable them to inhabit exposed habitats with nutrient-poor soils (46). As such, these would be expected to be more abundant in secondary peatland habitats recovering from fire disturbance compared to unburned, relatively undisturbed peat-swamp forests.

S4.1.3: Meta-analysis specification

Meta-analyses were specified within a Bayesian framework, implemented in rstan (47) called through R version 4.0.2 (48) using the package "brms" (49). Throughout, we use default uninformative priors for intercept and variance parameters drawn from a Student's T distribution (degrees of freedom=3; mu=-0.5/0; scale parameter=2.5). We ran four Markov chains of 10,000 iterations, discarding an initial burn-in phase of 5,000 and thinned by a rate of 5, resulting in 3,000 posterior draws for each parameter. Convergence was assessed through visual inspection of trace plots and numerically using the Gelman-Rubin statistic (values <1.1 indicate parameter convergence: (50)). We consider statistical differences between burn treatments and unburned controls to be substantial if the 95% Bayesian Credible Interval (BCI; 2.5th and 97.5th percentile of the posterior distribution) did not overlap zero and moderate if the 75% BCI (12.5th and 87.5th percentile of the posterior distribution) did not overlap zero.

S4.2: Estimating proportional changes in ecological components

To quantify proportional changes in ecosystem properties and biodiversity characteristics, we constructed generalized linear mixed-effects models (GLMMs) for each ecological component:

$$Ecological\ component_j = \alpha_{fire\ treatment}$$

where $\alpha_{fire\ treatment}$ denotes a fixed intercept for each fire treatment. For datasets sharing a common response variable (vegetation density and vegetation cover) or taxonomically aggregated groups (Odonata and herpetofauna), models contained an additional hierarchical component whereby effects for individual ecosystem elements or taxonomic units were drawn as random effects from a common distribution. This approach allowed inference at multiple ecological scales, while improving estimation precision for model parameters. We modelled continuous responses (daily maximum temperature, aboveground biomass, canopy height, avian-focused soundscapes) as realisations of a Gaussian distribution (truncated at zero for datasets that could not take negative values), count data (species richness/abundance for all taxonomic groups) as realisations of a Poisson distribution (or negative binomial distribution to address overdispersion) on the log scale, and proportional data (vegetation cover) as realisations of a beta distribution on the logit scale.

S4.3: Time-series modelling

S4.3.1: Data processing

For ecosystem property data, we defined seasons to align with the monthly sampling frequency. Biodiversity datasets were collected using temporally-replicated surveys, therefore we constructed species-specific detection histories for each taxonomic group by pooling detection/non-detection data into discrete sampling occasions according to site visits (butterflies and fish) or aggregated trap nights when monitoring was continuous (terrestrial vertebrates; 7 trap nights = 1 sampling occasion). Across taxonomic groups, we only generated detection histories for species with more than 25 detections throughout the duration of sampling, resulting in a total of 52 species for analysis. For butterflies and fish, which are relatively short-lived and have a fast reproductive rate, sampling occasions were grouped into one-month seasons. For terrestrial vertebrates, seasons were restricted to three-month intervals to ensure demographic closure, which is a fundamental assumption of our modelling approach (51). Three-month seasons were defined based around months of peak fire activity (August-October).

S4.3.2: GLMM model structure for ecosystem property data

To quantify temporal trends in ecosystem properties relative to historical megafire events, we constructed models of the form:

$$Ecosystem\ property_{j,t} = \beta_t + \gamma_j$$

where each ecosystem property is described using a season-specific intercept (β_t) and a spatial random effect term at the scale of sampling location (γ_j). We modelled ecosystem properties as realizations of Gaussian (river pH, leaf-fall) and beta (tree leaf flush and reproductive phenology) distributions, with the latter specified on the logit scale.

S4.3.3: Occupancy modelling framework for biodiversity data

Our occupancy framework specified the true occurrence state ($z_{j,t,i}$) of species *i* at sampling location *j*, and season *t* as a binary variable (1 = present; 0 = not detected), described as the realisation of a Bernoulli process:

$$z_{j,t,i} \sim Bernoulli(\psi_{j.t,i})$$

where $\psi_{j.t,i}$ describes the probability that a species occurs at a given sampling location during a given sampling season. As true occurrence is only partially observed, we introduce a second Bernoulli process to account for imperfect detection:

$$y_{j,t,i,k} \mid z_{j,t,i} \sim Bernoulli(p_{j,t,i,k} \cdot z_{j,t,i})$$

where $y_{j,t,i,k}$ is the observed detection/non-detection data for species *i*, at sampling location *j*, within season *t* and secondary sampling occasion *k*, and $p_{j,t,i,k}$ represents the probability of detecting the species given that it occupies the sampling location during a given season.

To examine biodiversity responses to the temporal signature of fire, we specified hierarchical multispecies occupancy models, of the form:

$$logit(\psi_{j,t,i}) = \beta_{i,t} + \gamma_{i,j}$$

$$logit(p_{j,t,i,k}) = \alpha_{o,i,t} + \alpha_{1,i,t} Effort_{j,t}$$

where occupancy and detection were modelled using random intercepts for each species and season ($\beta_{i,t}$ and $\alpha_{oi,t}$ respectively), with detection also expressed as a function of season-specific survey effort

("Effort"). We implemented a random walk prior on the species-specific occupancy intercepts, which specified the initial occupancy status for each species ($\beta_{i,1}$) using an uninformative normal distribution expressing no prior knowledge about species occurrence in season one. Subsequent seasonal trends were drawn from independent normal distributions parameterised using the mean occupancy estimate from the previous season. This introduced the expectation that temporal trends in occupancy are likely to be similar between subsequent seasons, with the degree of similarity controlled by the inter-season variance (52). The random walk prior was implemented using:

$$\beta_{i,1} \sim Normal(\mu_{\beta i}, 10^4)$$

$$\beta_{i,t>1} \sim Normal(\mu_{\beta i,t-1}, \sigma^2_{\beta i})$$

where the variance parameter $\sigma^2_{\beta i}$controls the degree to which mean occupancy for each species and season deviates from that of the previous season.

S4.3.5: Candidate model structure

For each ecological component, we specified three candidate models that introduced different covariate structures to understand how peatland ecosystem dynamics are impacted by different aspects of the fire regime. Throughout, temporal GLMMs adopted a bivariate specification to avoid overparameterization, resulting in five candidate models for ecosystem properties:

- Null model: explores temporal trends only using random intercepts, specified for GLMMs and occupancy models as described in sections S4.3.2 and S4.3.3.
- Fire properties model: incorporated all elements of the null model but introduced covariates to capture the impact of fire frequency and intensity on ecological components. We express fire intensity as the sum of fire radiative power for all fire detections within the neighbourhood of the sampling location.

$$Ecosystem\ property_{j,t} = \beta_{0,t} + \beta_1 Fire\ frequency_{j,t} + \gamma_j$$

$$Ecosystem\ property_{j,t} = \beta_{0,t} + \beta_1 Fire\ intensity_{j,t} + \gamma_j$$

$$logit(\psi_{j,t,i}) = \beta_{0,i,t} + \beta_{1,i} Fire\ frequency_{j,t} + \beta_{2,i} Fire\ intensity_{j,t} + \gamma_{i,j}$$

- Spatio-temporal proximity model: incorporated all the elements of the null model but introduced covariates to understand how distance in space and time mediates megafire impacts. Specifically we estimated how distance (km) from the perimeter of the most recent fire, and time (months) since the megafire event, affected ecological components.

$$Ecosystem\ property_{j,t} = \beta_{0,t} + \beta_1 Dist_{j,t} + \gamma$$

$$Ecosystem\ property_{j,t} = \beta_{0,t} + \beta_1 Time_{j,t} + \gamma_j$$

$$logit(\psi_{j,t,i}) = \beta_{0,i,t} + \beta_{1,i} Distance_{j,t} + \beta_{2,i} Time_{j,t} + \gamma_{i,j}$$

S4.3.6: Time-series model specification and evaluation

All analyses conducted were specified within a Bayesian framework, implemented in JAGS (53) called through R version 4.0.2 (48) using jagsUI (54). Throughout, we use uninformative flat normal and wide uniform priors on slope/intercept and variance parameters, respectively, with the exception of standard deviation parameters in the occupancy models, which were specified using half-Cauchy hyperpriors to prevent boundary effects (52). For GLMMs/occupancy models, we ran three Markov chains of 75,000/150,000 iterations, discarding an initial burn-in phase of 25,000/50,000 and thinned by a rate of 50/100, resulting in 3,000 posterior draws for each parameter. Convergence was assessed through visual inspection of trace plots and numerically using the Gelman-Rubin statistic (values <1.1 indicate parameter convergence: (50). Model fit was assessed using Pearson χ^2 discrepancy statistic, where values between 0.05 and 0.95 indicate adequate model fit (55). Across all models, we consider statistical differences and associations to be substantial if the 95% Bayesian Credible Interval (BCI; 2.5th and 97.5th percentile of the posterior distribution) did not overlap zero and moderate if the 75% BCI (12.5th and 87.5th percentile of the posterior distribution) did not overlap zero. Unless stated otherwise, we present modelled outputs as posterior means and 95% BCIs. All post-hoc calculations were estimated using the full posterior distribution, to explicitly account for uncertainty.

Table S1: Summary details of ecological burn treatment and time-series datasets collated for assessing, respectively, direct and indirect fire impacts, plus post-fire recovery. Where relevant, the number of species detected and included within our analyses are listed in the Metric column. See Appendix S2 for full method descriptions.

Dataset	Metric (units) [no. species]	Data collection date range	Burn date (time since burn[1])	No. of megafire events	Data collection method (sampling frequency)
(a) Fire treatment data					
Ecosystem properties					
Microclimate	Maximum daily temperature (ºC)	Aug-Sep 2018	Old burn: Pre-1997 (20 yr 11 mo)[2] New burn: Oct 2015 (2 yr 10 mo)	-	Thermochron attached to passive acoustic monitoring unit (continuous, daily)
Vegetation cover	Grass (proportion)	Feb-May 2019	Old burn: Oct 2006 (12 y 4 mo) New burn: Oct 2015 (3 yr 4 mo)		Visual estimate within 10 x 10 m plot
	Fern (proportion)	Feb-May 2019	Old burn: Oct 2006 (12 y 4 mo) New burn: Oct 2015 (3 yr 4 mo)		Visual estimate within 10 x 10 m plot
	Canopy cover (proportion)	Feb-May 2019	Old burn: Oct 2006 (12 y 4 mo) New burn: Oct 2015 (3 yr 4 mo)		Visual estimate within 10 x 10 m plot
Vegetation density	Pitcher plants (counts $m^{2\ -1}$)	Feb-May 2019	Old burn: Oct 2006 (12 y 4 mo) New burn: Oct 2015 (3 yr 4 mo)		Count within 5 x 5 m nested vegetation plots
	Pandans (counts $m^{2\ -1}$)	Feb-May 2019	Old burn: Oct 2006 (12 y 4 mo) New burn: Oct 2015 (3 yr 4 mo)		Count within 5 x 5 m nested vegetation plots
	Lianas (counts $m^{2\ -1}$)	Feb-May 2019	Old burn: Oct 2006 (12 y 4 mo) New burn: Oct 2015 (3 yr 4 mo)		Count within 5 x 5 m nested vegetation plots
	Seedlings (counts $m^{2\ -1}$)	Feb-May 2019	Old burn: Oct 2006 (12 y 4 mo) New burn: Oct 2015 (3 yr 4 mo)		Count within 2 x 2 m sub-plots
	Saplings (counts $m^{2\ -1}$)	Feb-May 2019	Old burn: Oct 2006 (12 y 4 mo) New burn: Oct 2015 (3 yr 4 mo)		Count within 5 x 5 m nested vegetation plots

Dataset	Metric (units) [no. species]	Data collection date range	Burn date (time since burn[1])	No. of megafire events	Data collection method (sampling frequency)
	Trees (counts $m^{2\ -1}$)	Feb-May 2019	Old burn: Oct 2006 (12 y 4 mo)<br>New burn: Oct 2015 (3 yr 4 mo)		Count within 10 x 10 m vegetation plots
Forest structure	Mean canopy height (m)	Feb-May 2019	Old burn: Oct 2006 (12 y 4 mo)<br>New burn: Oct 2015 (3 yr 4 mo)		Visual estimates within 10 x 10 m vegetation plots
	Aboveground live biomass (t ha^{-1})	Feb-May 2019	Old burn: Oct 2006 (12 y 4 mo)<br>New burn: Oct 2015 (3 yr 4 mo)		Derived from measured tree diameters and heights within the 10 x 10 m plots, plus species-specific wood densities
Biodiversity characteristics					
Trees	Trees (species encounters/frequency) [45 spp.]	Feb-May 2019	Old burn: Oct 2006 (12 y 4 mo)	-	Forest inventory surveys within 10 x 10 m vegetation plots
Odonata	Anisoptera (species encounters/frequency) [22 spp.]	Apr 2017-Nov 2019	Old burn: Oct 2006 (10 y 6 mo)	-	Visual encounter surveys along line transects (monthly)
	Zygoptera (species encounters/frequency) [25 spp.]	Apr 2017-Nov 2019	Old burn: Oct 2006 (10 y 6 mo)	-	Visual encounter surveys along line transects (monthly)
Butterflies	Papilionoidea (species encounters/frequency) [16 spp.]	Nov 2018-Jul 2019	Old burn: N/A<br>New burn: Oct 2015 (3 yr 1 mo)	-	Fruit-baited canopy traps (five consecutive days per month)
Herpetofauna	Amphibia (species encounters/frequency) [6 spp.]	Aug-Sep 2021	Old burn: N/A<br>New burn: Oct 2015 (5 yr 10 mo)	-	Diurnal and nocturnal visual encounter surveys along line transects (three consecutive surveys per transect)
	Reptilia (species encounters/frequency) [12 spp.]	Aug-Sep 2021	Old burn: N/A<br>New burn: Oct 2015 (5 yr 10 mo)	-	Diurnal and nocturnal visual encounter surveys along line transects (three consecutive surveys per transect)
Avian-focused soundscapes	Acoustic Complexity Index (ACI)	Sep 2018-Aug 2019	Old burn: Pre-1997 (21 yr 0 mo)2<br>New burn: Oct 2015 (2 yr 11 mo)	-	Passive acoustic monitoring (continuous two-hour recordings, daily)
	Acoustic Diversity Index (ADI)	Sep 2018-Aug 2019	Old burn: Pre-1997 (21 yr 0 mo)2<br>New burn: Oct 2015 (2 yr 11 mo)	-	Passive acoustic monitoring (continuous two-hour recordings, daily)
	Bioacoustic Index (BIO)	Sep 2018-Aug 2019	Old burn: Pre-1997 (21 yr 0 mo)2<br>New burn: Oct 2015 (2 yr 11 mo)	-	Passive acoustic monitoring (continuous two-hour recordings, daily)
	Normalised Difference Soundscape Index (NDSI)	Sep 2018-Aug 2019	Old burn: Pre-1997 (21 yr 0 mo)2<br>New burn: Oct 2015 (2 yr 11 mo)	-	Passive acoustic monitoring (continuous two-hour recordings, daily)

(b) Time-series data					
Ecosystem properties					
Water quality	Acidity (pH)	Sep 2014-Dec 2015	-	2	pH metre (monthly)
Forest productivity	Leaf-fall (g cm$^{3\ -1}$)	Dec 2005--Jan 2018	-	4	Litter-fall traps (monthly)
Tree phenology	Leaf flush (proportion)	Sep 2003-Jan 2018	-	5	Nested vegetation plots (monthly)
	Flower expression (proportion)	Sep 2003-Jan 2018	-	5	Nested vegetation plots (monthly)
	Fruit expression (proportion)	Sep 2003-Jan 2018	-	5	Nested vegetation plots (monthly)
Biodiversity characteristics					
Butterflies	Papilionoidea (species) [28 spp.] encounters/frequency)	Jan 2012-Jan 2018	-	2	Fruit-baited canopy traps (monthly)
Fish	River fish (species encounters/frequency) [38 spp.]	Sep 2014-Dec 2015	-	2	Baited river traps (monthly)
Terrestrial vertebrates	Ground-dwelling birds (species encounters/frequency) [13 spp.]	May 2008-Jan 2020	-	4	Camera-traps (continuous)
	Medium-large terrestrial mammals (species encounters/frequency) [27 spp.]	May 2008-Jan 2020	-	4	Camera-traps (continuous)

1. Indicates the duration from the last burn date to the time data collection began for each dataset.
2. Exact date of last burning unknown, but reports from long-term local residents indicate that the area has not burnt after the major 1997-98 fires in the region, which is supported by field observations. For the purposes of calculating the recovery period difference from new to old burn, this area is therefore conservatively assumed to have last burned towards the end of this dry season; i.e., in October 1997.

Table S2: Scale optimisation summary for Bayesian generalized linear mixed-effects models and multispecies occupancy models employed to assess the temporal response of ecological components to characteristics of fire regimes. Scale optimisation was conducted to identify the spatial extents that best characterised responses to fire impacts for each time-series dataset and select the best performing fire intensity metric. We selected optimal spatial scales/fire intensity metrics based on Watanabe-Akaike Information Criterion (WAIC), with the lowest values indicating the best overall model (presented in bold).

Ecological component	Covariate	Scale	BPV	WAIC
Ecosystem properties				
**pH**	Fire frequency	1000m	0.505	-503.13
		1500m	0.504	-504.88
		2000m	0.508	-504.33
		2500m	0.524	-504.22
		**3000m**	**0.505**	**-506.71**
		3500m	0.513	-503.26
		4000m	0.526	-504.58
		4500m	0.512	-504.69
		5000m	0.522	-502.63
	Fire radiative power: maximum	1000m	0.512	-504.19
		1500m	0.512	-503.99
		**2000m**	**0.513**	**-505.65**
		2500m	0.526	-503.5
		3000m	0.497	-504.38
		3500m	0.51	-505.23
		4000m	0.529	-504.63
		4500m	0.508	-504.12
		5000m	0.52	-504.2
	Fire radiative power: mean	1000m	0.515	-501.9
		1500m	0.506	-505.59
		2000m	0.513	-505.16
		2500m	0.518	-506.22
		3000m	0.514	-505.56
		**3500m**	**0.521**	**-507.3**
		4000m	0.509	-503.35
		4500m	0.52	-505.63
		5000m	0.519	-502.87
	Fire radiative power: standard deviation	1000m	0.509	-504.44
		1500m	0.512	-505.59
		2000m	0.511	-504.09
		2500m	0.512	-503.43
		3000m	0.51	-503.92
		3500m	0.513	-502.88
		4000m	0.498	-506.47
		4500m	0.522	-505.34
		**5000m**	**0.517**	**-507.39**
	Fire radiative power: sum	1000m	0.522	-502.29
		1500m	0.521	-499.54
		2000m	0.492	-501.65
		2500m	0.505	-503.15

**Ecological component**	**Covariate**	**Scale**	**BPV**	**WAIC**
		3000m	0.502	-501.01
		3500m	0.511	-503.03
		4000m	0.509	-504.57
		**4500m**	**0.507**	**-508.43**
		5000m	0.504	-502.58
**Litter-fall**	Fire frequency	2500m	0.511	-711.33
		**3000m**	**0.508**	**-727.77**
		3500m	0.496	-695.82
		4000m	0.504	-684.76
		4500m	0.497	-665.18
		5000m	0.501	-634.24
	Fire radiative power: maximum	**2500m**	**0.497**	**-731.83**
		3000m	0.518	-695.7
		3500m	0.515	-701.93
		4000m	0.502	-704.99
		4500m	0.493	-697.64
		5000m	0.501	-685.48
	Fire radiative power: mean	2500m	0.496	-723.75
		3000m	0.503	-701.61
		**3500m**	**0.508**	**-725.5**
		4000m	0.506	-686.97
		4500m	0.505	-632.8
		5000m	0.506	-645.72
	Fire radiative power: standard deviation	2500m	0.511	-711.72
		3000m	0.504	-724.67
		**3500m**	**0.493**	**-735.69**
		4000m	0.504	-702.3
		4500m	0.511	-670.95
		5000m	0.494	-696.61
	Fire radiative power: sum	**2500m**	**0.515**	**-733.36**
		3000m	0.509	-708.6
		3500m	0.507	-706.7
		4000m	0.501	-704.42
		4500m	0.516	-727.91
		5000m	0.516	-704.94
**Phenology: leaves**	Fire frequency	3000m	0.508	6738.71
		3500m	0.513	6736.02
		4000m	0.498	6741.94
		**4500m**	**0.509**	**6735.77**
		5000m	0.494	6754.28
	Fire radiative power: maximum	**3000m**	**0.511**	**6724.65**
		3500m	0.491	6734.82
		4000m	0.535	6733.38
		4500m	0.507	6728.38
		5000m	0.49	6747.03
	Fire radiative power: mean	3000m	0.501	6738.58
		3500m	0.501	6727.77
		**4000m**	**0.498**	**6721.3**
		4500m	0.508	6737.47
		5000m	0.509	6740.35
	Fire radiative power: standard deviation	3000m	0.5	6735.45
		3500m	0.501	6737.43
		4000m	0.513	6737.25
		**4500m**	**0.512**	**6731.48**
		5000m	0.498	6736.42
	Fire radiative power: sum	**3000m**	**0.48**	**6724.52**
		3500m	0.505	6734.94

Ecological component	Covariate	Scale	BPV	WAIC
		4000m	0.513	6743.63
		4500m	0.509	6734.61
		5000m	0.485	6733.84
**Phenology: flowers**	Fire frequency	3000m	0.522	4312.32
		3500m	0.503	4335.16
		**4000m**	**0.504**	**4303.05**
		4500m	0.5	4308.03
		5000m	0.493	4312.35
	Fire radiative power: maximum	3000m	0.507	4308.5
		3500m	0.506	4313.01
		**4000m**	**0.504**	**4300.43**
		4500m	0.505	4314.25
		5000m	0.499	4307.83
	Fire radiative power: mean	3000m	0.518	4312.99
		3500m	0.501	4318.42
		4000m	0.498	4315.09
		**4500m**	**0.485**	**4306.92**
		5000m	0.494	4314.83
	Fire radiative power: standard deviation	**3000m**	**0.514**	**4304.49**
		3500m	0.501	4314.04
		4000m	0.494	4310.48
		4500m	0.499	4317.51
		5000m	0.514	4307.95
	Fire radiative power: sum	3000m	0.503	4310.54
		3500m	0.519	4300.08
		4000m	0.51	4308.68
		**4500m**	**0.512**	**4297.7**
		5000m	0.497	4312.95
**Phenology: fruit**	Fire frequency	**3000m**	**0.503**	**4155.36**
		3500m	0.509	4156.67
		4000m	0.497	4166.07
		4500m	0.489	4169.22
		5000m	0.511	4160.89
	Fire radiative power: maximum	3000m	0.496	4165
		**3500m**	**0.515**	**4153.12**
		4000m	0.499	4171.45
		4500m	0.5	4161.41
		5000m	0.506	4167.1
	Fire radiative power: mean	**3000m**	**0.518**	**4152.88**
		3500m	0.495	4162.99
		4000m	0.516	4159.01
		4500m	0.507	4157.16
		5000m	0.502	4165.58
	Fire radiative power: standard deviation	**3000m**	**0.511**	**4155.52**
		3500m	0.497	4158.29
		4000m	0.515	4168.65
		4500m	0.512	4160.64
		5000m	0.511	4167.12
	Fire radiative power: sum	3000m	0.506	4165.32
		3500m	0.519	4162.56
		4000m	0.501	4160.82
		4500m	0.489	4170.4
		**5000m**	**0.495**	**4159.74**
**Biodiversity**				
**Butterflies**	Fire frequency	1000m	0.445	17380.58
		1500m	0.437	17369.04
		2000m	0.429	17372.77

Ecological component	Covariate	Scale	BPV	WAIC
		2500m	0.423	17370.74
		3000m	0.431	17374.45
		**3500m**	**0.419**	**17368.14**
		4000m	0.423	17373.09
		4500m	0.441	17374.77
		5000m	0.438	17381.01
	Fire radiative power: maximum	1000m	0.434	17377.82
		1500m	0.461	17383
		2000m	0.431	17369.14
		2500m	0.432	17372.2
		**3000m**	**0.426**	**17361.65**
		3500m	0.426	17383.2
		4000m	0.437	17371.28
		4500m	0.448	17379.9
		5000m	0.425	17379.04
	Fire radiative power: mean	1000m	0.442	17377.79
		1500m	0.433	17379.58
		2000m	0.431	17374.83
		2500m	0.417	17374.49
		3000m	0.435	17377.51
		3500m	0.443	17376.31
		4000m	0.434	17379.28
		**4500m**	**0.426**	**17372.26**
		5000m	0.427	17382.35
	Fire radiative power: standard deviation	1000m	0.429	17381.55
		1500m	0.435	17380.48
		2000m	0.449	17385.23
		2500m	0.427	17374.59
		3000m	0.449	17375.82
		3500m	0.435	17374.23
		4000m	0.444	17377.79
		**4500m**	**0.428**	**17372.65**
		5000m	0.413	17375.45
	Fire radiative power: sum	1000m	0.421	17374.63
		1500m	0.43	17377.76
		2000m	0.436	17372.61
		2500m	0.446	17375.01
		**3000m**	**0.419**	**17359.39**
		3500m	0.446	17382.79
		4000m	0.432	17376.76
		4500m	0.432	17378.55
		5000m	0.425	17377.94
	Distance to fire	**50m**	**0.432**	**17371.77**
		250m	0.435	17374.24
		500m	0.434	17378.25
**Fish**	Fire frequency	1000m	0.41	11090.07
		1500m	0.418	11089.3
		2000m	0.413	11105.78
		2500m	0.412	11096.27
		**3000m**	**0.404**	**11082.93**
		3500m	0.41	11093.86
		4000m	0.413	11094.79
		4500m	0.409	11094.76
		5000m	0.417	11091.44
	Fire radiative power: maximum	1000m	0.409	11089.33
		1500m	0.4	11082.34
		2000m	0.396	11092.41
		2500m	0.41	11075.64

Ecological component	Covariate	Scale	BPV	WAIC
		**3000m**	**0.411**	**11072.88**
		3500m	0.399	11087.56
		4000m	0.417	11086.79
		4500m	0.406	11089.77
		5000m	0.397	11086.35
	Fire radiative power: mean	1000m	0.422	11082.78
		1500m	0.399	11077.27
		2000m	0.403	11090.8
		2500m	0.417	11088.15
		3000m	0.411	11081.38
		3500m	0.388	11089.36
		4000m	0.4	11091.11
		**4500m**	**0.416**	**11071.86**
		5000m	0.433	11077.59
	Fire radiative power: standard deviation	**1000m**	**0.418**	**11047.46**
		1500m	0.413	11087.21
		2000m	0.399	11089.34
		2500m	0.42	11093.59
		3000m	0.398	11073.78
		3500m	0.423	11083.61
		4000m	0.406	11085.11
		4500m	0.437	11176.45
		5000m	0.419	11094.28
	Fire radiative power: sum	1000m	0.419	11084.4
		1500m	0.405	11094.94
		2000m	0.421	11080.44
		2500m	0.404	11075.69
		**3000m**	**0.409**	**11071.68**
		3500m	0.414	11076.77
		4000m	0.417	11083.7
		4500m	0.414	11078.45
		5000m	0.421	11074.99
	Distance to fire	50m	0.537	11266.43
		250m	0.399	11142.38
		**500m**	**0.402**	**11078.48**
**Birds**	Fire frequency	1000m	0.536	1200.17
		1500m	0.54	1199.67
		2000m	0.544	1207
		2500m	0.548	1189.11
		3000m	0.55	1234.11
		3500m	0.518	1221.3
		**4000m**	**0.532**	**1188.29**
		4500m	0.551	1216.62
		5000m	0.539	1261.13
	Fire radiative power: maximum	1000m	0.552	1174.1
		1500m	0.525	1198.38
		2000m	0.53	1206.13
		2500m	0.544	1182.88
		3000m	0.541	1175.15
		3500m	0.538	1198.92
		**4000m**	**0.526**	**1162**
		4500m	0.544	1175.01
		5000m	0.541	1193.66
	Fire radiative power: mean	1000m	0.559	1184.7
		1500m	0.544	1179.81
		2000m	0.524	1191.78
		2500m	0.548	1185.45
		3000m	0.554	1206.35

Ecological component	Covariate	Scale	BPV	WAIC
		3500m	0.535	1210.16
		4000m	0.558	1194.23
		**4500m**	**0.539**	**1170.26**
		5000m	0.547	1178.18
	Fire radiative power: standard deviation	1000m	0.537	1209.23
		1500m	0.528	1163.15
		2000m	0.538	1191.57
		2500m	0.547	1181.69
		3000m	0.528	1186.09
		3500m	0.543	1186.25
		4000m	0.546	1206.25
		4500m	0.531	1183.5
		**5000m**	**0.541**	**1155.49**
	Fire radiative power: sum	1000m	0.536	1185.33
		1500m	0.55	1185.15
		2000m	0.554	1204.97
		2500m	0.527	1176.66
		3000m	0.542	1190.92
		3500m	0.54	1168.28
		**4000m**	**0.541**	**1166.26**
		4500m	0.532	1191.25
		5000m	0.537	1196.48
	Distance to fire	50m	0.534	1175.03
		**250m**	**0.54**	**1158.51**
		500m	0.538	1202.55
**Mammals**	Fire frequency	1000m	0.519	11524.34
		1500m	0.516	11512.36
		2000m	0.514	11517.42
		2500m	0.5	11528.45
		3000m	0.527	11527.36
		3500m	0.507	11519.09
		4000m	0.504	11514.64
		**4500m**	**0.509**	**11503.59**
		5000m	0.515	11510.93
	Fire radiative power: maximum	1000m	0.501	11518.54
		1500m	0.523	11518.52
		2000m	0.518	11523.94
		2500m	0.494	11514.91
		3000m	0.499	11694.87
		3500m	0.508	11501.26
		**4000m**	**0.5**	**11475.03**
		4500m	0.512	11510.43
		5000m	0.499	11503.76
	Fire radiative power: mean	1000m	0.526	11516.31
		1500m	0.505	11512.94
		2000m	0.499	11519.84
		2500m	0.508	11598.6
		3000m	0.516	11517.01
		3500m	0.52	11506.1
		4000m	0.513	11506.42
		4500m	0.513	11518.9
		**5000m**	**0.515**	**11494.77**
	Fire radiative power: standard deviation	1000m	0.511	11643.06
		1500m	0.51	11520.53
		2000m	0.509	11522.16
		2500m	0.506	11514.09
		3000m	0.507	11521.71
		**3500m**	**0.512**	**11486.02**

**Ecological component**	**Covariate**	**Scale**	**BPV**	**WAIC**
		4000m	0.497	11495.65
		4500m	0.498	11495.78
		5000m	0.506	11509.6
	Fire radiative power: sum	1000m	0.506	11505.48
		1500m	0.491	11501.19
		2000m	0.504	11522.19
		**2500m**	**0.492**	**11487.68**
		3000m	0.502	11499.98
		3500m	0.523	11491.76
		4000m	0.509	11503.06
		4500m	0.507	11517.08
		5000m	0.526	11517.25
	Distance to fire	50m	0.506	11490.71
		250m	0.488	11523.64
		**500m**	**0.525**	**11485.3**

Table S3: Details of group assignments for modelling temporal responses to fire regimes. Taxa were aggregated based on IUCN threat status (Non-threatened: Least Concern LC, Near-threatened NT; Threatened: Vulnerable VU, Endangered EN, Critically Endangered CR; analyzed for mammals and ground-dwelling birds only), feeding guilds (PanTHERIA, (56): mammals only) and commercially valuable species ((57, 58): fish only).

Taxon/Species	IUCN status	Threat status	Feeding guild	Commercial status
Ground-dwelling birds				
Babbler sp.	LC	Non-threatened	-	-
Crestless fireback	VU	Threatened	-	-
Greater coucal	LC	Non-threatened	-	-
Storm’s stork	EN	Threatened	-	-
Medium-large mammals				
Bearded pig	VU	Threatened	Omnivore	-
Bornean yellow muntjac	LC	Non-threatened	Herbivore	-
Flat-headed cat	EN	Threatened	Carnivore	-
Leopard cat	LC	Non-threatened	Carnivore	-
Malay civet	LC	Non-threatened	Carnivore	-
Marbled cat	VU	Threatened	Carnivore	-
Mouse-deer sp.	LC	Non-threatened	Frugivore	-
Orangutan	CR	Threatened	Frugivore	-
Otter civet	EN	Threatened	Carnivore	-
Pig-tailed macaque	VU	Threatened	Frugivore	-
Short-tailed mongoose	LC	Non-threatened	Carnivore	-
Small-toothed palm civet	LC	Non-threatened	Omnivore	-
Sun bear	VU	Threatened	Omnivore	-
Sunda clouded leopard	VU	Threatened	Carnivore	-
Yellow-throated marten	LC	Non-threatened	Carnivore	-
Fish				
Belontia hasselti	LC	Non-threatened	-	Valuable
Clarias sp.	-	-	-	Valuable
Cyclocheilichthys janthochir	LC	Non-threatened	-	Valuable
Desmopuntius foerschi	NT	Non-threatened	-	Non-valuable
Desmopuntius hexazona	LC	Non-threatened	-	Non-valuable
Desmopuntius johorensis	LC	Non-threatened	-	Non-valuable
Eirmotus sp.	-	-	-	Non-valuable
Kryptopterus sp.	-	-	-	Non-valuable
Leiocassis micropogon	LC	Non-threatened	-	Non-valuable
Luciocephalus aura	EN	Threatened	-	Non-valuable
Luciocephalus pulcher	LC	Non-threatened	-	Non-valuable
Macrognathus maculatus	LC	Non-threatened	-	Non-valuable
Mystus olyroides	NT	Non-threatened	-	Valuable
Nandus nebulosus	LC	Non-threatened	-	Valuable
Osteochilus spilurus	LC	Non-threatened	-	Non-valuable
Pristolepis grootii	LC	Non-threatened	-	Non-valuable
Pseudeutropius moolenburghae	LC	Non-threatened	-	Non-valuable
Rasbora cephalotaenia	LC	Non-threatened	-	Non-valuable
Sphaerichthys acrostoma	VU	Threatened	-	Non-valuable
Striuntius lineatus	LC	Non-threatened	-	Non-valuable
Channa sp.	-	-	-	Valuable

Table S4: Model fit and selection statistics for Bayesian generalized linear mixed-effects models and multispecies occupancy models employed to assess the temporal response of ecological components to different characteristics of fire regimes. We specified three candidate models for each ecological component: null model, accounting for temporal trends only; fire properties model, to understand how fire frequency and intensity shape peatland ecosystem dynamics; spatio-temporal proximity model, to assess how distance (km) from the perimeter of the most recent megafire and time (months) since the megafire event affected ecological components. Throughout, temporal GLMMs adopted a bivariate specification for the fire frequency and spatio-temporal proximity models to avoid overparameterization, resulting in five candidate models for ecosystem properties. Model fit was assessed using Bayesian P values (BPV), where 0.05 < BPV < 0.95 indicates adequate model fit and BPV = 0.5 reflects perfect model fit. Models are presented in descending order of performance using Watanabe-Akaike Information Criterion (WAIC). ΔWAIC indicates variation in WAIC relative to the top-ranking model and quantifies the strength of evidence between competing models. Models with ΔWAIC < 2 were considered to have substantial statistical support, while 2 < ΔWAIC < 8 were considered to have moderate support.

Ecological component	Model	BPV	WAIC	ΔWAIC
Ecosystem properties				
**River pH**	Fire intensity	0.507	-508.43	0.00
	Time since megafire	0.511	-507.75	0.68
	Fire frequency	0.505	-504.88	3.55
	Distance to megafire	0.512	-503.37	5.05
	Null	0.497	-501.63	6.80
**Leaf-fall**	Fire intensity	0.515	-733.36	0.00
	Fire frequency	0.508	-727.77	5.59
	Time since megafire	0.508	-723.77	9.59
	Distance to megafire	0.502	-720.58	12.78
	Null	0.504	-717.69	15.67
**Phenology: leaves**	Time since megafire	0.505	6727.31	0.00
	Distance to megafire	0.521	6732.41	5.11
	Null	0.514	6733.11	5.80
	Fire intensity	0.509	6734.61	7.30
	Fire frequency	0.498	6741.94	14.63
**Phenology: flowers**	Distance to megafire	0.512	4297.70	0.00
	Fire intensity	0.504	4303.05	5.36
	Null	0.518	4309.21	11.51
	Fire frequency	0.526	4311.82	14.12
	Time since megafire	0.509	4324.04	26.34
**Phenology: fruit**	Fire intensity	0.512	4160.64	0.00
	Time since megafire	0.512	4162.25	1.61

Ecological component	Model	BPV	WAIC	ΔWAIC
	Fire frequency	0.497	4166.07	5.43
	Null	0.503	4167.96	7.32
	Distance to megafire	0.506	4181.79	21.15
Biodiversity				
**Butterflies**	Spatio-temporal proximity to megafire	0.421	16954.99	0.00
	Fire properties	0.423	16960.25	5.26
	Null	0.427	16974.72	19.73
**Fish**	Fire properties	0.417	10900.75	0.00
	Spatio-temporal proximity to megafire	0.429	10902.15	1.41
	Null	0.436	10903.62	2.87
**Birds**	Fire properties	0.540	1146.54	0.00
	Spatio-temporal proximity to megafire	0.540	1149.48	2.94
	Null	0.556	1150.78	4.24
**Mammals**	Fire properties	0.518	10962.13	0.00
	Spatio-temporal proximity to megafire	0.508	10968.17	6.04
	Null	0.516	10969.02	6.89

Table S5: Indonesian research permit details for data collection and use.

Dataset	Time period	Indonesian data (co-) owner/s (whole period)	International data co-owners (selected periods: SIP #)
Fire treatment data			
Microclimate	2018	Darmae Nasir[1] & Muhammad Ali Imron	Mark Harrison (2019-22: 12/E5/E5.4/SIP/2020, 7/E5/E5.4/SIP.EXT/2021 & 43/SIP.EXT/IV/FR/12/2021)
Vegetation cover, density & forest structure; Trees	2019	Ici Kulu	William Healey (2019: 46/E5/E5.4/SIP/2019)
Odonata	2017-19	Ici Kulu	Brendan Holly (2018-19: 365/SIP/FRP/E5/Dit.KI/X/2018)
Butterflies	2018-19	Ici Kulu	William Healey (2019: 46/E5/E5.4/SIP/2019)
Herpetofauna	2021	Hastin Ambar Asti & Muhammad Ali Imron	N/A
Avian focused soundscapes	2018-19	Darmae Nasir[1] & Muhammad Ali Imron	Mark Harrison (2019-22: 12/E5/E5.4/SIP/2020, 7/E5/E5.4/SIP.EXT/2021 & 43/SIP.EXT/IV/FR/12/2021)
Time-series data			
Tree phenology	2003-18	Darmae Nasir[2] & Muhammad Ali Imron	Mark Harrison (2019-22: 12/E5/E5.4/SIP/2020, 7/E5/E5.4/SIP.EXT/2021 & 43/SIP.EXT/IV/FR/12/2021; 2012-14: 245/SIP/FRP/SM/VII/2012 & 47/EXT/SIP/FRP/SM/VI/2013; 2010-11: 0018/EXT/FRP/SM/III/2010, 0039/EXT/FRP/SM/IX/2010 & 0141/SIP/FRP/VI/2011; 2005-07, LIPI: 3547/SU/KS/2005 & 06603/SU/KS/2006)<br>Nicholas Marchant (2011-13: 274/SIP/FRP/SM/VII/2011 & 58/EXT/SIP/FRP/SM/VII/2012)<br>Helen Morrogh-Bernard (2011-12: 012/SIP/FRP/I/2011; LIPI, 2003-05, permit no. unknown)
Forest productivity	2005-18	Darmae Nasir[2] & Muhammad Ali Imron	Mark Harrison (2019-22: 12/E5/E5.4/SIP/2020, 7/E5/E5.4/SIP.EXT/2021 & 43/SIP.EXT/IV/FR/12/2021; 2012-14: 245/SIP/FRP/SM/VII/2012 & 47/EXT/SIP/FRP/SM/VI/2013; 2010-11: 0018/EXT/FRP/SM/III/2010, 0039/EXT/FRP/SM/IX/2010 & 0141/SIP/FRP/VI/2011; 2005-07, LIPI: 3547/SU/KS/2005 & 06603/SU/KS/2006)<br>Nicholas Marchant (2011-13: 274/SIP/FRP/SM/VII/2011 & 58/EXT/SIP/FRP/SM/VII/2012)<br>Helen Morrogh-Bernard (2011-12: 012/SIP/FRP/I/2011; LIPI, 2003-05, permit no. unknown)

Dataset	Time period	Indonesian data (co-) owner/s (whole period)	International data co-owners (selected periods: SIP #)
Butterflies	2012-18	Darmae Nasir[2] & Muhammad Ali Imron	Mark Harrison (2019-22: 12/E5/E5.4/SIP/2020, 7/E5/E5.4/SIP.EXT/2021 & 43/SIP.EXT/IV/FR/12/2021; 2012-14: 245/SIP/FRP/SM/VII/2012 & 47/EXT/SIP/FRP/SM/VI/2013)<br>Joana Aragay Soler (2015-16: 131/SIP/FRP/SM/V/ 2015 & 24/EXT/SI P/FRP/E5/Dit.KI/IV/2016)<br>Nicholas Marchant (2011-13: 274/SIP/FRP/SM/VII/2011 & 58/EXT/SIP/FRP/SM/VII/2012)<br>Nicholas Boyd (2011-12, SIP no. unknown)
Water quality & fish	2014-15	Yulentine	Sara Thornton (2015-16: 34/EXT/SIP/FRP/SM/VII/2015 & 01/EXT/SIP/FRP/E5/Dit.KI/I/2016)
Terrestrial vertebrates	2009-18	Darmae Nasir[2] & Jito Sugardjito	Susan Cheyne (2019-21: 203/E5/E5.4/SIP/2019; 2015: 360/SIP/FRP/E5/Dit.KI/IX/2015 & 217/EXT/VSIP/FRP/SVI/2015; 2013-14: 217/SIP/FRP/SM/VI/2013 & 217/VSIP/FRP/SVI/2014; 2011-12: 26/EXT/SIP/FRP/SM/VI/2011 & 45/EXT/SIP/FRP/SM/VI/2012; 2009-10: 850D/FRP/SM/VI/2009 & 925D/FRP/SM/VI/2009)<br>Karen Jeffers (2016-18: 47/EXT/SIP/FRP/E5/Dit.KI/IX/2016, 217/EXT/VSIP/FRP/SVI/2016, 55/EXT/SIP/FRP/E5/Dit.KI/VIII/2017 & 260/SIP/FRP/E5/Dit.KI/IX/2018)<br>Bernat Ripoll Capilla (2011-12: 050/SIP/FRP/I/2011)<br>Joana Aragay Soler (2011-12: 185xx & 16/EXT/SI P/FRP/SM/II/2012)
Advanced data analysis			
All	N/A	Jatna Supriatna	Matthew Struebig (2021-2022: 42/TU.B5.4/SIP/VIII/2021)<br>Nicolas Deere (2021-2022: 42/TU.B5.4/SIP/VIII/2021)

1. Indicates datasets previously (co-)owned by Yusurum Jagau (deceased), with UPR data (co-)ownership transferred to Darmae Nasir;
2. Indicates datasets previously (co-)owned by Yusurum Jagau & Suwido H. Limon (both deceased), with UPR data (co-)ownership transferred to Darmae Nasir.

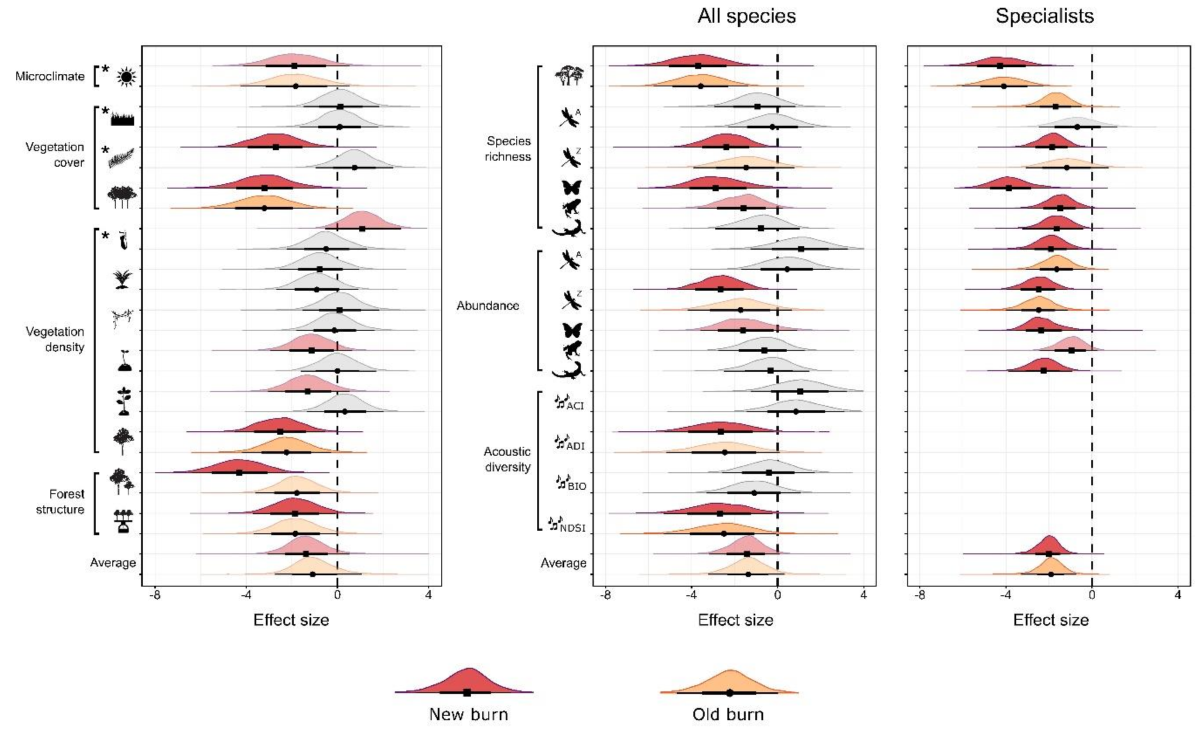

Figure S1: Standardized mean differences between burn treatments and unburned controls (vertical dashed black line) for peatland ecosystem properties (top to bottom: maximum daily temperature, grass/fern/canopy cover, pitcher/pandan/liana.seedling/sapling/tree density, canopy height and aboveground biomass), and biodiversity characteristics for both all species and forest specialists (top to bottom: species richness and abundance of trees/dragonflies/damselflies/butterflies/ amphibians/reptiles, Acoustic Complexity Index, Acoustic Diversity Index, Bioacoustic Index, Normalized Difference Soundscape Index). Burn treatments captured "new burn" areas burned in the latest megafire event in 2015 (point symbols and red hues) and "old burn" areas recovering from fire activity dating back to 2006 (square symbols and orange hues). The "Overall" response combines effect sizes for both ecosystem properties and biodiversity across individual datasets to provide a quantitative, aggregate summary of how fires broadly impact the ecosystem's abiotic and biotic components. All results are posterior mean values (symbols) with 75% Bayesian Credible Intervals (BCI; thick black horizontal lines) and 95% BCI (thin black horizontal lines). The concentration of values across the posterior distribution is visualized above posterior summaries and colour-coded to reflect burn, with an asterisks by a variable indicating instances where effect size directions have been reversed to more clearly indicate positive or negative fire impacts (see Appendix S4.1.2 for details). Substantial statistical differences between treatments and the unburned control are indicated by darker hues for color-coded

distributions and moderate differences by lighter hues for color-coded distributions, with non-influential parameters presented in gray (see Appendix S4.1.3 for details).

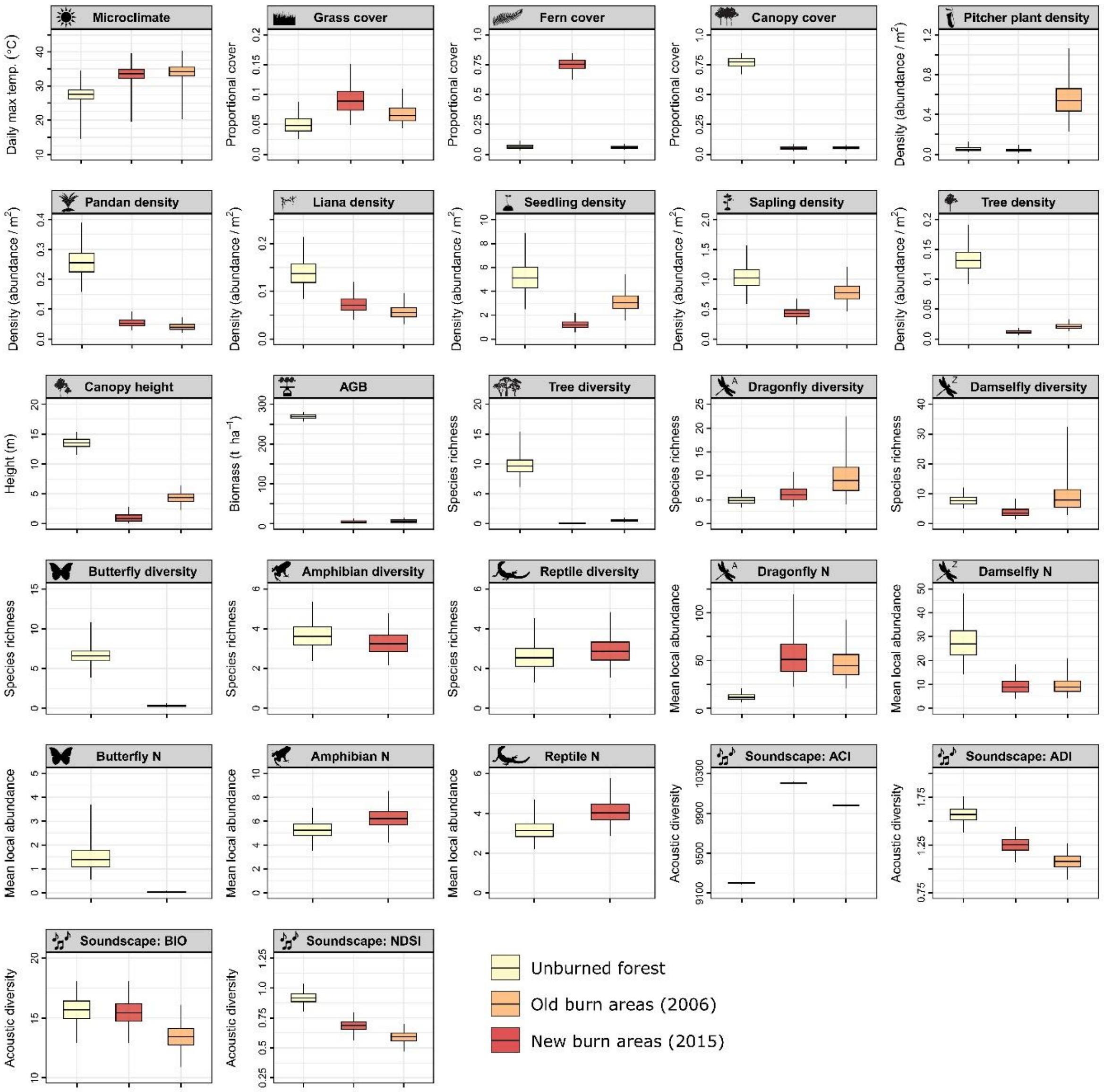

Figure S2: Modelled averages for ecosystem properties and biodiversity (all species) in burn treatments relative to unburned forest controls (yellow boxes). Burn treatments captured areas subjected to a recent fire event in 2015 ("new burn"; red hues) and those recovering from periods of fire activity dating back to 2006 ("old burn"; orange hues). Boxes denote the 25th, 50th (horizontal black line in the middle of each box) and 75th percentiles of the posterior distribution, while vertical black lines express uncertainty using as 95% Bayesian Credible Intervals. Abbreviations: AGB = above-ground biomass; N = abundance; ACI = Acoustic Complexity Index; ADI = Acoustic Diversity Index; BIO = Bioacoustic Index; NDSI = Normalised Difference Soundscape Index. For definitions of symbols, see Figure 2 in main text.

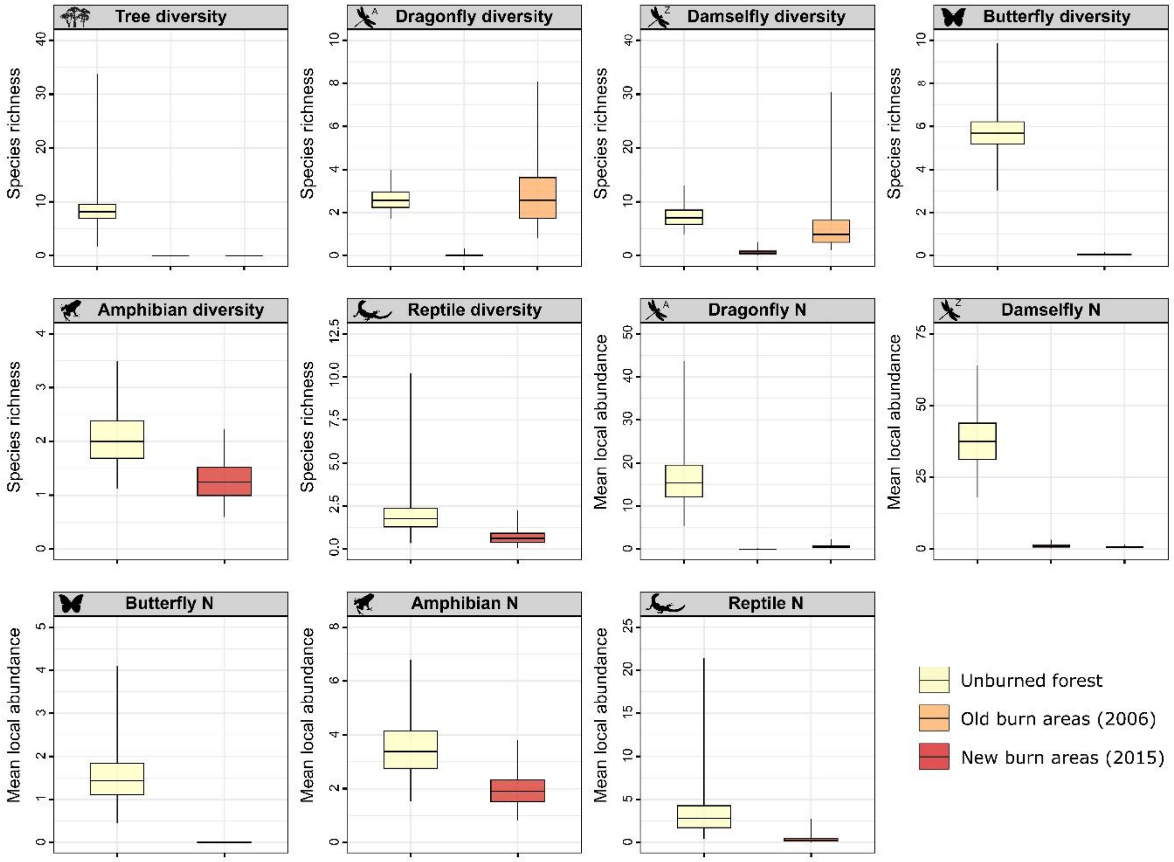

Figure S3: Modelled averages for forest specialist biodiversity in burn treatments relative to unburned forest controls (yellow boxes). Burn treatments captured areas subjected to a recent fire event in 2015 (“new burn”; red hues) and those recovering from periods of fire activity dating back to 2006 (“old burn”; orange hues). Boxes denote the 25th, 50th (horizontal black line in the middle of each box) and 75th percentiles of the posterior distribution, while vertical black lines express uncertainty using as 95% Bayesian Credible Intervals. For definitions of symbols, see Figure 2 in main text.

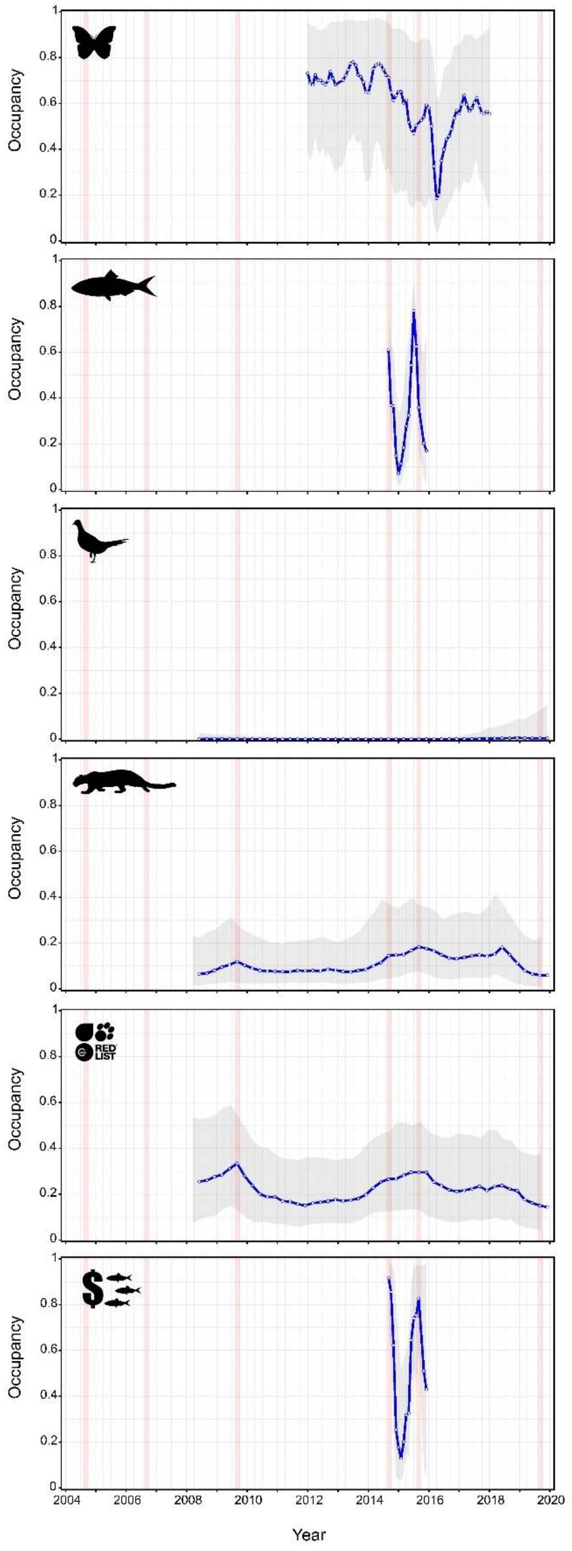

Figure S4: Temporal trends (blue lines) demonstrated by forest specialist species (right panel; occupancy of butterflies, fish, ground-dwelling birds, medium-large terrestrial mammals, IUCN-threatened vertebrates, commercially valuable fish) in response to multiple megafire events (red vertical lines) across a 16-year timeframe (January 2004 to January 2020). Throughout, occupancy reflects the

probability that the species is present in the study landscape, where a value of zero indicates that the species is completely absent during and a value of one confirms that the species was present during the observed timepoint. We present temporal summaries as posterior means of season-specific intercept terms (hollow blue points) and express uncertainty using 95% Bayesian Credible Intervals (gray ribbons).

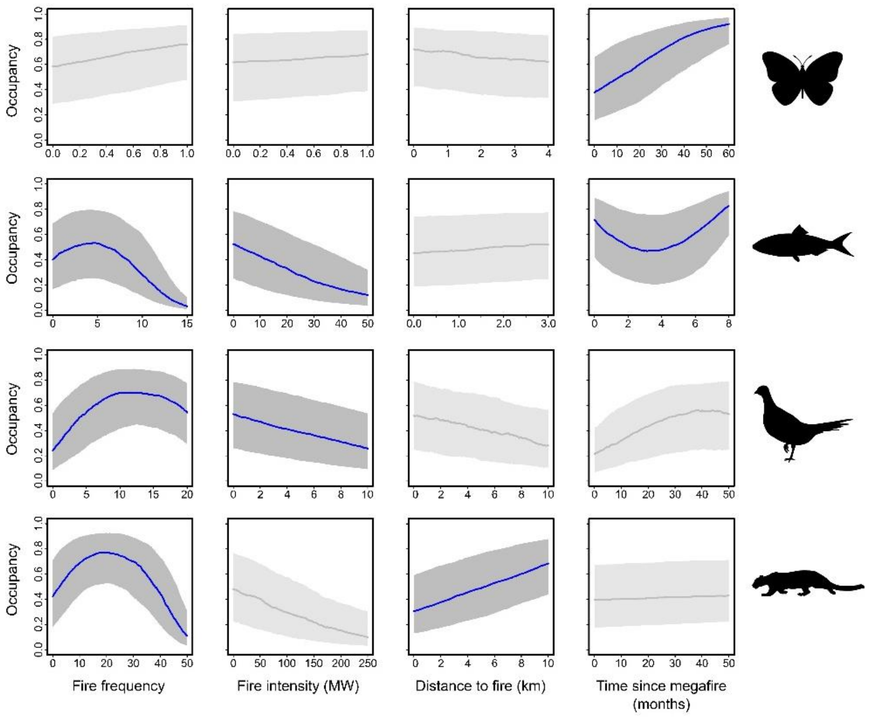

Figure S5: Forest specialist species responses to fire properties (fire frequency, fire radiative power) and the spatiotemporal proximity to extreme thermal events (distance from megafire, time since last megafire). Occupancy reflects the probability that the species is present in the study landscape, where a value of zero indicates that the species is completely absent during and a value of one confirms that the species was present during the observed time point. Solid blue lines denote the mean of the posterior distribution while grey ribbons denote uncertainty, expressed using 95% Bayesian Credible Intervals. Non-influential parameters are presented in light gray. For definition of symbols, see Figure 3.

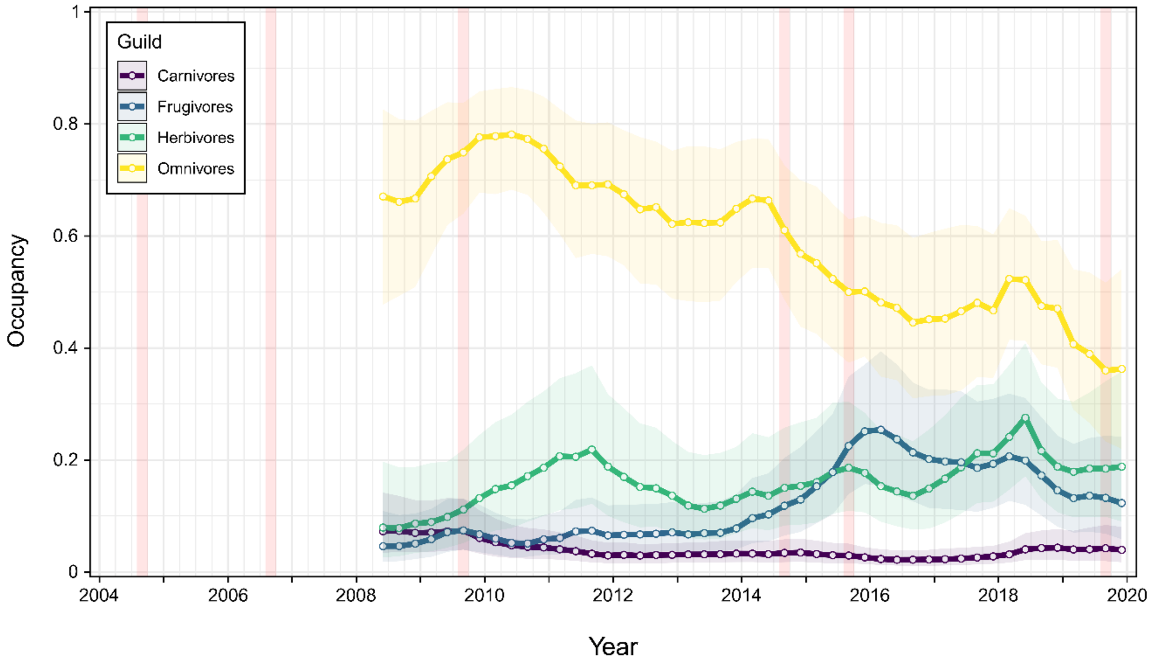

Figure S6: Temporal trends (blue and colour-coded lines) in occurrence for four distinct mammal guilds, in response to multiple megafire events (red vertical lines) across a 16-year timeframe (January 2004 to January 2020). Temporal summaries are posterior means of season-specific intercept terms (hollow points) and express uncertainty using 95% Bayesian Credible Intervals (colour-coded ribbons).

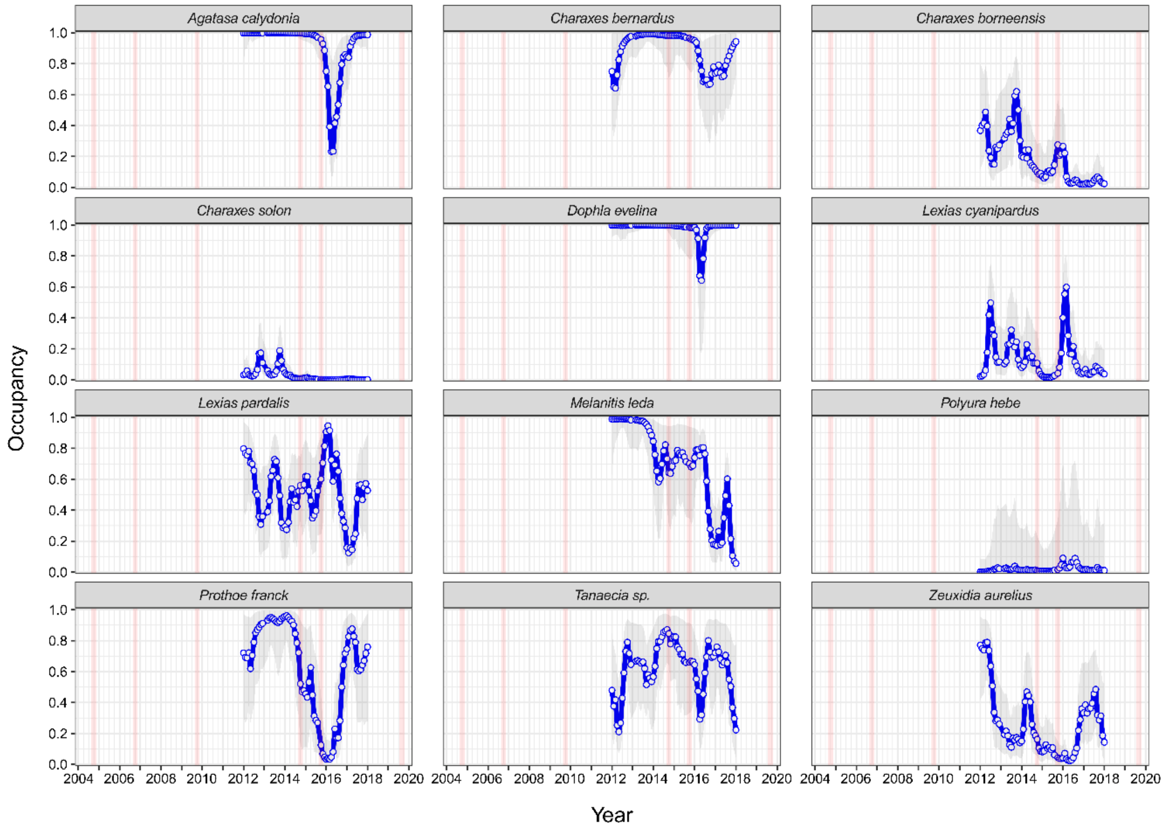

Figure S7: Temporal trends (blue lines) in occurrence for 12 butterfly species in response to multiple megafire events (red vertical lines) across a 16-year timeframe (January 2004 to January 2020). Occupancy reflects the probability that the species is present in the study landscape, where a value of zero indicates that the species is completely absent during and a value of one confirms that the species was present during the observed time point. Temporal summaries ae presented as posterior means of season-specific intercept terms (hollow points) and express uncertainty using 95% Bayesian Credible Intervals (gray ribbons).

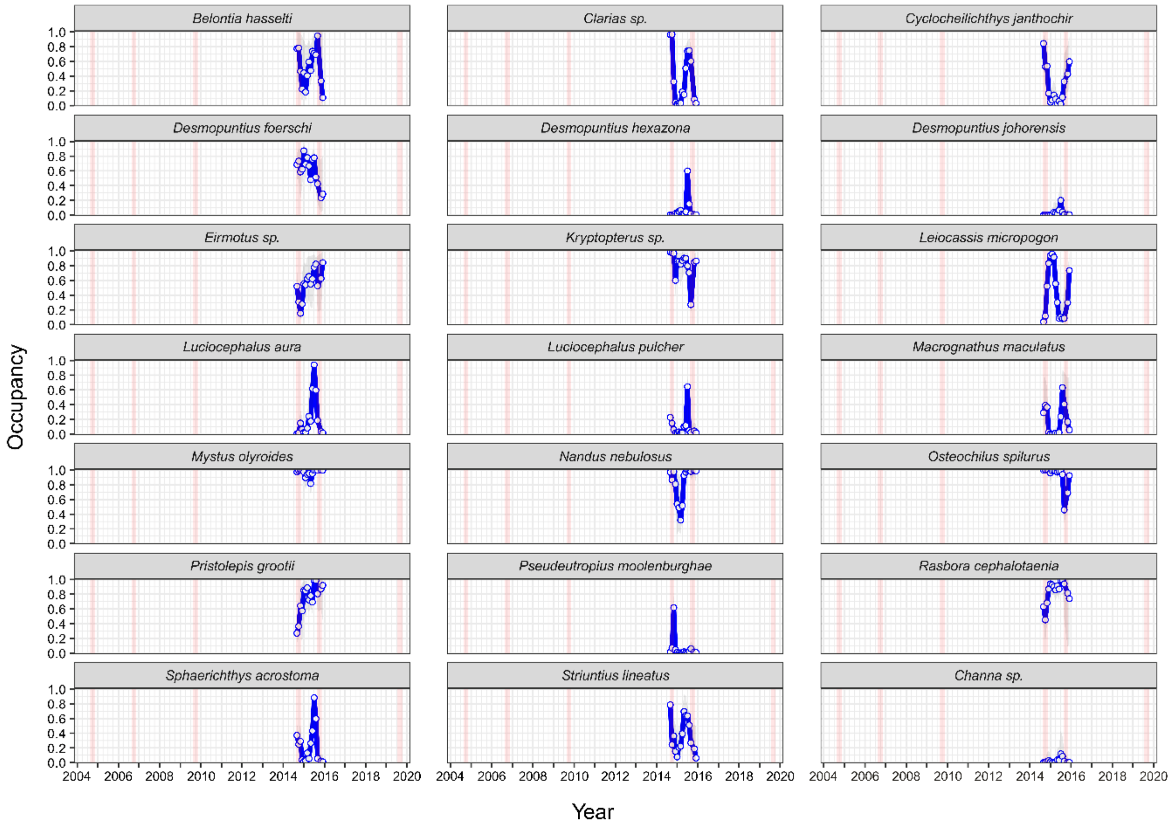

Figure S8: Temporal trends (blue lines) in occurrence for 21 fish species in response to multiple megafire events (red vertical lines) across a 16-year timeframe (January 2004 to January 2020). Occupancy reflects the probability that the species is present in the study landscape, where a value of zero indicates that the species is completely absent during and a value of one confirms that the species was present during the observed time point. Temporal summaries are presented as posterior means of season-specific intercept terms (hollow points) and express uncertainty using 95% Bayesian Credible Intervals (gray ribbons).

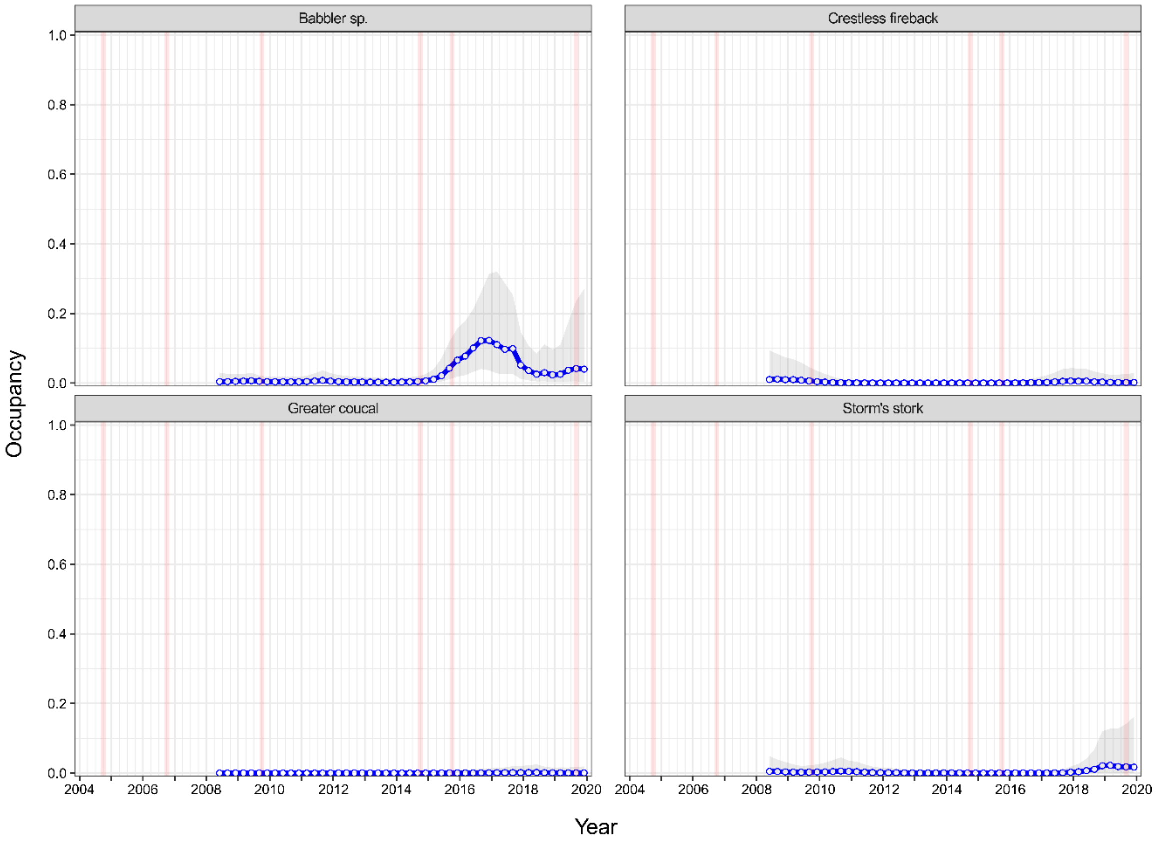

Figure S9: Temporal trends (blue lines) in occurrence for four ground-dwelling bird species in response to multiple megafire events (red vertical lines) across a 16-year timeframe (January 2004 to January 2020). Occupancy reflects the probability that the species is present in the study landscape, where a value of zero indicates that the species is completely absent during and a value of one confirms that the species was present during the observed time point. Temporal summaries are presented as posterior means of season-specific intercept terms (hollow points) and express uncertainty using 95% Bayesian Credible Intervals (gray ribbons).

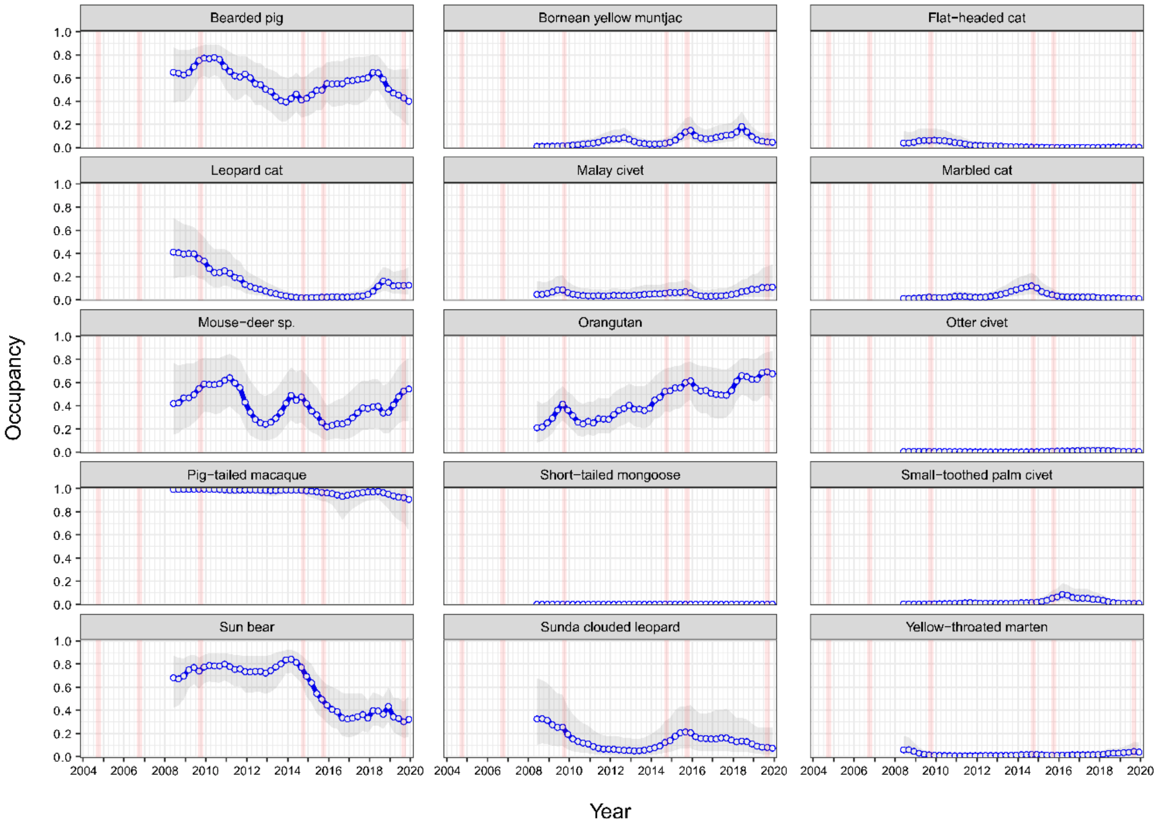

Figure S10: Temporal trends (blue lines) in occurrence for 15 medium-large mammal species in response to multiple megafire events (red vertical lines) across a 16-year timeframe (January 2004 to January 2020). Occupancy reflects the probability that the species is present in the study landscape, where a value of zero indicates that the species is completely absent during and a value of one confirms that the species was present during the observed time point. Temporal summaries are presented as posterior means of season-specific intercept terms (hollow points) and express uncertainty using 95% Bayesian Credible Intervals (gray ribbons).

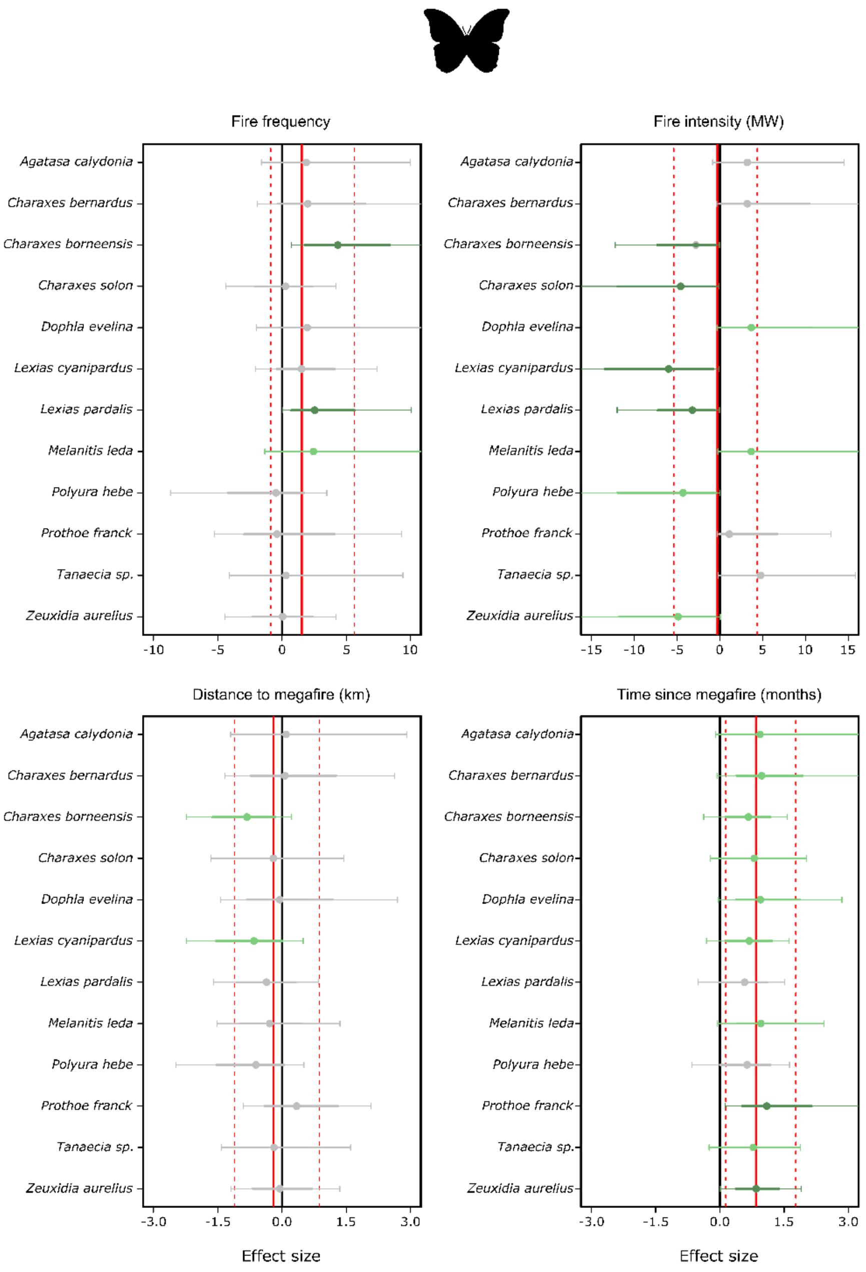

Figure S11: Relative influence of fire properties (fire frequency, fire radiative power) and spatio-temporal proximity to thermal events (distance from fire, time since last fire) on butterfly occurrence represented by 12 species. Effect sizes are expressed as the mean of the posterior distribution (community: solid red vertical line; species: points) and uncertainty is denoted using 75% and 95% Bayesian Credible Intervals (BCIs; community: dashed vertical red lines, 95% BCI only; species: thick and thin horizontal lines respectively). Posterior summaries are colour-coded to reflect the extent of influence (gray = no effect; light tones = moderate effect, 75% BCI does not overlap zero; dark tones = substantial effect, 95% BCI does not overlap zero; zero represented by solid black line).

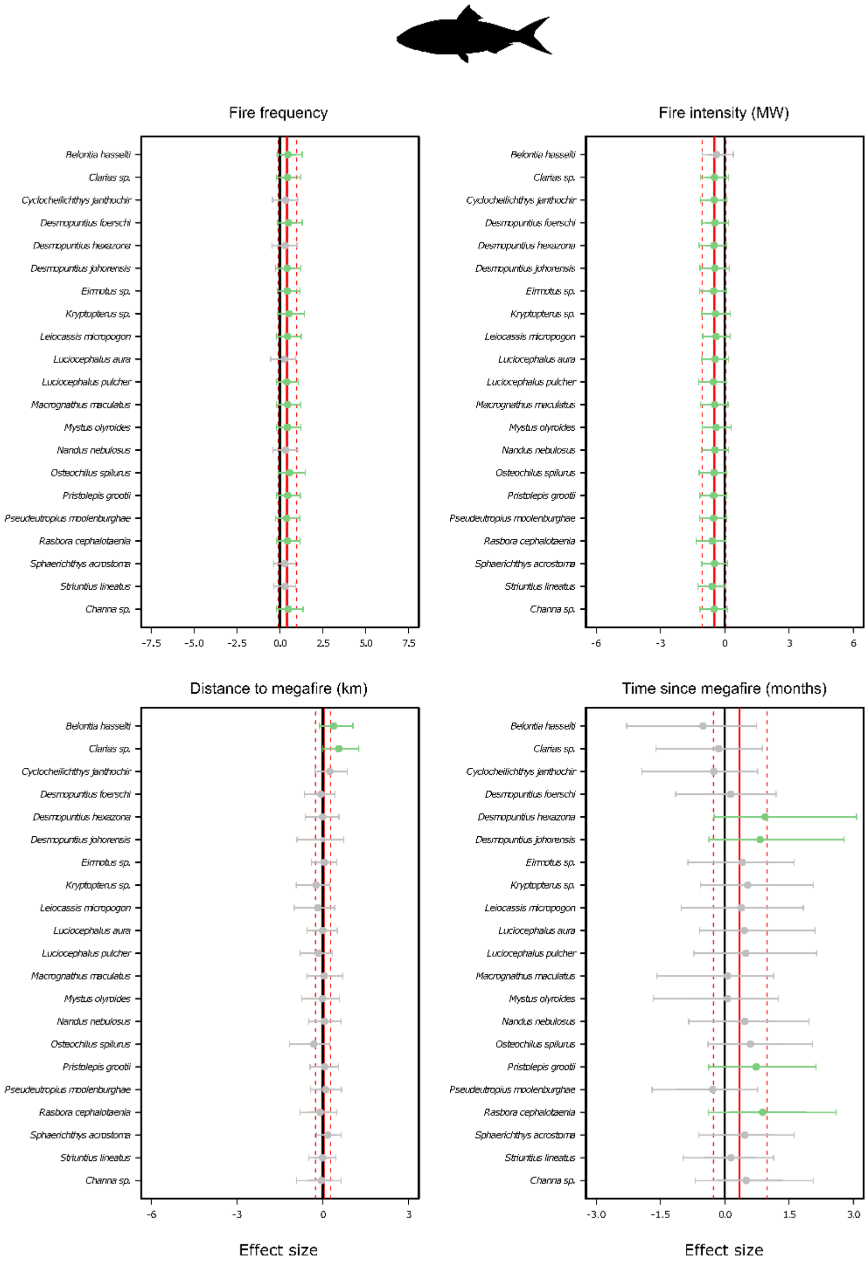

Figure S12: Relative influence of fire properties (fire frequency, fire radiative power) and spatio-temporal proximity to thermal events (distance from fire, time since last fire) on fish occurrence represented by 21 species. Effect sizes are expressed as the mean of the posterior distribution (community: solid red vertical line; species: points) and uncertainty is denoted using 75% and 95% Bayesian Credible Intervals (BCIs; community: dashed vertical red lines, 95% BCI only; species: thick and thin horizontal lines respectively). Posterior summaries are colour-coded to reflect the extent of influence (gray = no effect; light tones = moderate effect, 75% BCI does not overlap zero; dark tones = substantial effect, 95% BCI does not overlap zero; zero represented by solid black line).

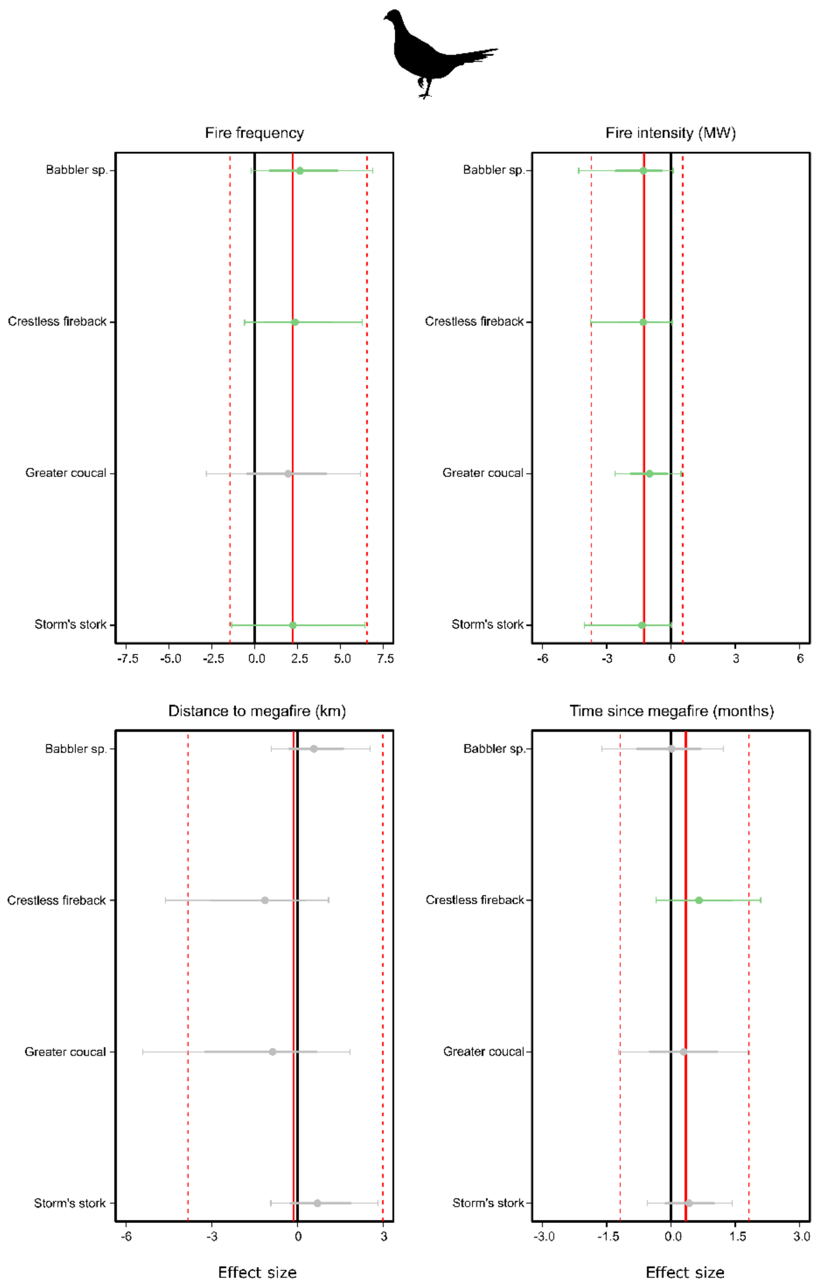

Figure S13: Relative influence of fire properties (fire frequency, fire radiative power) and spatiotemporal proximity to thermal events (distance from fire, time since last fire) on ground-dwelling bird occurrence represented by four species. Effect sizes are expressed as the mean of the posterior distribution (community: solid red vertical line; species: points) and uncertainty is denoted using 75% and 95% Bayesian Credible Intervals (BCIs; community: dashed vertical red lines, 95% BCI only; species: thick and thin horizontal lines respectively). Posterior summaries are colour-coded to reflect the extent of influence (gray = no effect; light tones = moderate effect, 75% BCI does not overlap zero; dark tones = substantial effect, 95% BCI does not overlap zero; zero represented by solid black line).

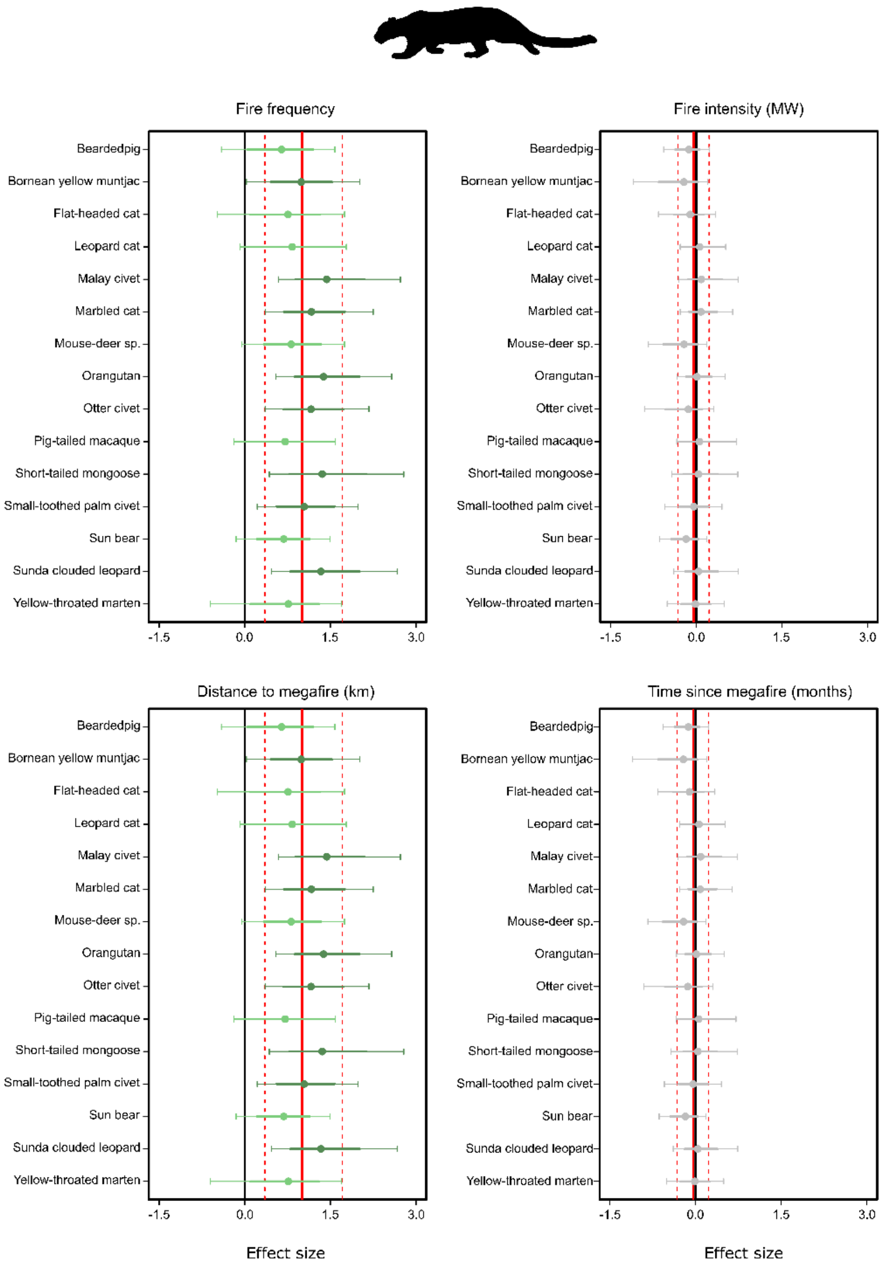

Figure S14: Relative influence of fire properties (fire frequency, fire radiative power) and spatio-temporal proximity to thermal events (distance from fire, time since last fire) on medium-large mammal occurrence represented by 15 species. Effect sizes are expressed as the mean of the posterior distribution (community: solid red vertical line; species: points) and uncertainty is denoted using 75% and 95% Bayesian Credible Intervals (BCIs; community: dashed vertical red lines, 95% BCI only; species: thick and thin horizontal lines respectively). Posterior summaries are colour-coded to reflect the extent of influence (gray = no effect; light tones = moderate effect, 75% BCI does not overlap zero; dark tones = substantial effect, 95% BCI does not overlap zero; zero represented by solid black line).

References Cited in Supplementary Information

1. J. G. Pausas, J. E. Keeley, Wildfires and global change. *Frontiers in Ecology and the Environment* **19**, 387-395 (2021).
2. L. Kiely *et al.*, Air quality and health impacts of vegetation and peat fires in Equatorial Asia during 2004–2015. *Environmental Research Letters* **15**, 094054 (2020).
3. N. Yulianti *et al.*, The linkage of El Niño-induced peat fires and its relation to current haze condition in Central Kalimantan. *Journal of Wetlands Environmental Management* **8**, 100-116 (2020).
4. S. J. Husson *et al.*, Biodiversity of the Sabangau tropical peat-swamp forest, Indonesian Borneo. *Mires and Peat* **22**, (05) 1-50 (2018).
5. A. E. Zanne *et al.*, Global wood density database. *Dryad* DOI: 10.5061/dryad.234 (2009).
6. J. Chave *et al.*, Improved allometric models to estimate the aboveground biomass of tropical trees. *Global Change Biology* **20**, 3177-3190 (2014).
7. D. F. R. Cleary *et al.*, Diversity and community composition of butterflies and odonates in an ENSO-induced fire affected habitat mosaic: a case study from East Kalimantan, Indonesia. *Oikos* **105**, 426-446 (2004).
8. S. H. Luke *et al.*, The impacts of habitat disturbance on adult and larval dragonflies (Odonata) in rainforest streams in Sabah, Malaysian Borneo. *Freshwater Biology* **62**, 491-506 (2017).
9. A. Cordero-Rivera, R. Stoks, "Mark-recapture studies and demography" in Dragonflies and Damselflies: Model Organisms for Ecological and Evolutionary Research, A. Cordero-Rivera, Ed. (Oxford University Press, Oxford, UK, 2008), pp. 7-20.
10. A. Dolný, F. Harabiš, D. Bárta, S. Lhota, P. Drozd, Aquatic insects indicate terrestrial habitat degradation: changes in taxonomical structure and functional diversity of dragonflies in tropical rainforest of East Kalimantan. *Tropical Zoology* **25**, 141-157 (2012).
11. J. M. B. Oliveira-Junior, L. Juen, The Zygoptera/Anisoptera ratio (Insecta: Odonata): a new tool for habitat alterations assessment in Amazonian streams. *Neotropical Entomology* **48**, 552-560 (2019).
12. P. R. Houlihan, M. E. Harrison, S. M. Cheyne, Impacts of forest gaps on butterfly diversity in a Bornean peat-swamp forest. *Journal of Asia-Pacific Entomology* **16**, 67-73 (2013).

13. N. C. Marchant *et al.*, 'Random-flight' dispersal in tropical fruit-feeding butterflies? High mobility, long lifespans and no home ranges. *Ecological Entomology* **40**, 696-706 (2015).
14. A. Purwanto *et al.* Good Practice Guidelines: Butterfly Canopy Trapping. (Orangutan Tropical Peatland Project, Palangka Raya, Indonesia, 2015).
15. B. C. Pijanowski *et al.*, Soundscape ecology: the science of sound in the landscape. *BioScience* **61**, 203-216 (2011).
16. T. Bradfer-Lawrence *et al.*, Guidelines for the use of acoustic indices in environmental research. *Methods in Ecology and Evolution* **10**, 1796-1807 (2019).
17. L. J. Villanueva-Rivera, B. C. Pijanowski, Soundecology: soundscape Ecology. R package version 1.3.3. (2018)
18. T. Bradfer-Lawrence, N. Bunnefeld, N. Gardner, S. G. Willis, D. H. Dent, Rapid assessment of avian species richness and abundance using acoustic indices. *Ecological Indicators* **115**, 106400 (2020).
19. N. T. Boelman, G. P. Asner, P. J. Hart, R. E. Martin, Multi-trophic invasion resistance in Hawaii: Bioacoustics, field surveys, and airborne remote sensing. *Ecological Applications* **17**, 2137-2144 (2007).
20. C. Mammides, E. Goodale, S. K. Dayananda, L. Kang, J. Chen, Do acoustic indices correlate with bird diversity? Insights from two biodiverse regions in Yunnan Province, south China. *Ecological Indicators* **82**, 470-477 (2017).
21. A. Gasc *et al.*, Soundscapes reveal disturbance impacts: biophonic response to wildfire in the Sonoran Desert Sky Islands. *Landscape Ecology* **33**, 1399-1415 (2018).
22. B. P. Y.-H. Lee, Z. G. Davies, M. J. Struebig, Smoke pollution disrupted biodiversity during the 2015 El Niño fires in Southeast Asia. *Environmental Research Letters* **12**, 094022 (2017).
23. S. A. Thornton, Dudin, S. E. Page, C. Upton, M. E. Harrison, Peatland fish of Sebangau, Borneo: Diversity, monitoring and conservation. *Mires and Peat* **22**, (04) 1-25 (2018).
24. M. C. Gupta, A. K. M. Ghouse, Effects of coal-smoke pollutants from different sources on the growth, chlorophyll content, stem anatomy and cuticular traits of *Euphorbia hirta* L. *Environmental Pollution* **47**, 221-229 (1987).
25. T. T. Kozlowski, Impacts of air pollution on forest ecosystems. *BioScience* **30**, 88-93 (1980).

26. M. E. Harrison, S. M. Cheyne, Y. Sulistiyanto, J. O. Rieley, "Biological effects of smoke from dry-season fires in non-burnt areas of the Sabangau peat-swamp forest, Central Kalimantan, Indonesia" in *Carbon-Climate-Human Interaction on Tropical Peatland. Proceedings of The International Symposium and Workshop on Tropical Peatland, Yogyakarta, 27-29 August 2007, EU CARBOPEAT and RESTORPEAT Partnership, Gadjah Mada University, Indonesia and University of Leicester, United Kingdom,* J. O. Rieley, C. J. Banks, B. Radjagukguk, Eds. (2007).

27. M. E. Harrison, B. Ripoll Capilla, S. A. Thornton, M. E. Cattau, S. E. Page, "Impacts of the 2015 fire season on peat-swamp forest biodiversity in Indonesian Borneo" in *Peatlands in Harmony - Agriculture, Industry & Nature. Proceedings of the 15th International Peat Congress: Oral Presentations. 15-19 August 2016, Sarawak, Malaysia*. (International Peat Society, 2016), pp. 713-717.

28. M. E. Harrison *et al.*, Disparity in onset timing and frequency of new flowering and fruiting events in two Bornean peat-swamp forests. *Biotropica* **48**, 188-197 (2016).

29. M. E. Harrison, H. C. Morrogh-Bernard, D. J. Chivers, Orangutan energetics and the influence of fruit availability in the non-masting peat-swamp forest of Sabangau, Indonesian Borneo. *International Journal of Primatology* **31**, 585-607 (2010).

30. A. Wilting, F. Fischer, S. Abu Bakar, K. E. Linsenmair, Clouded leopards, the secretive top-carnivore of South-East Asian rainforests: their distribution, status and conservation needs in Sabah, Malaysia. *BMC Ecology* **6**, 16 (2006).

31. I. T. C. Wibisono, A. Dohong, *Technical Guidance for Peatland Revegetation* (Badan Restorasi Gambut, Jakarta, Indonesia, 2017), 105 pp.

32. L. L. B. Graham, W. Giesen, S. E. Page, A common-sense approach to tropical peat swamp forest restoration in Southeast Asia. *Restoration Ecology* **25**, 312-321 (2017).

33. A. G. Orr, "Odonata in Bornean tropical rain forest formations: diversity, endemicity and implications for conservation management" in *Forests and Dragonflies,* A. C. Rivera, Ed. (Fourth WDA International Symposium of Odonatology, Pontevedra, Spain, 2006), pp. 51-78.

34. R. A. Dow, M. J. Silvius, Results of an Odonata survey carried out in the peatlands of Central Kalimantan, Indonesia, in 2012. *Faunistic Studies in South-East Asian and Pacific Island Odonata* **7**, 1-37 (2014).

35. T. Hirowatari, H. Makihara, Sugiarto, Effects of fires on butterfly assemblages in lowland dipterocarp forest in East Kalimantan. *Entomological Science* **10**, 113-127 (2007).

36. N. J. Deere *et al.*, Riparian buffers can help mitigate biodiversity declines in oil palm agriculture. *Frontiers in Ecology and the Environment* **20**, 459-466 (2022).

37. K. A. Wilson *et al.*, Conserving biodiversity in production landscapes. *Ecological Applications* **20**, 1721-1732 (2010).

38. T. C. Hsieh, K. H. Ma, A. Chao, iNEXT: an R package for rarefaction and extrapolation of species diversity (Hill numbers). *Methods in Ecology and Evolution* **7**, 1451-1456 (2016).

39. A. Chao, L. Jost, Coverage-based rarefaction and extrapolation: standardizing samples by completeness rather than size. *Ecology* **93**, 2533-2547 (2012).

40. M. Borenstein, L. Hedges, J. Higgins, H. Rothstein, *Introduction to Meta-Analysis* (John Wiley & Sons, Chicester, UK, 2011).

41. D. G. Bonett, Meta-analytic interval estimation for standardized and unstandardized mean differences. *Psychological Methods* **14**, 225-238 (2009).

42. R. B. de Andrade, J. K. Balch, J. Y. O. Carreira, P. M. Brando, A. V. L. Freitas, The impacts of recurrent fires on diversity of fruit-feeding butterflies in a south-eastern Amazon forest. *Journal of Tropical Ecology* **33**, 22-32 (2017).

43. A. G. dos Anjos, M. Solé, M. Benchimol, Fire effects on anurans: What we know so far? *Forest Ecology and Management* **495**, 119338 (2021).

44. S. Page *et al.*, Restoration ecology of lowland tropical peatlands in Southeast Asia: current knowledge and future research directions. *Ecosystems* **12**, 888-905 (2009).

45. G. V. Blackham, E. L. Webb, R. T. Corlett, Natural regeneration in a degraded tropical peatland, Central Kalimantan, Indonesia: Implications for forest restoration. *Forest Ecology and Management* **324**, 8-15 (2014).

46. J. H. Adam, C. C. Wilcock, M. D. Swaine, The ecology and distribution of Bornean "*Nepenthes*". *Journal of Tropical Forest Science* **5**, 13-25 (1992).

47. Stan Development Team, "RStan: the R interface to Stan." R package version 2.21.8. (https://mc-stan.org/, 2023).

48. R Core Team, R: A language and environment for statistical computing. R Foundation for Statistical Computing. (https://www.R-project.org/, Vienna, Austria, 2022).

49. P.-C. Bürkner, brms: An R Package for Bayesian Multilevel Models Using Stan. *Journal of Statistical Software* **80**, 1 - 28 (2017).

50. A. Gelman, D. B. Rubin, Inference from iterative simulation using multiple sequences. *Statistical Science* **7**, 457-472 (1992).

51. C. T. Rota, R. J. Fletcher Jr, R. M. Dorazio, M. G. Betts, Occupancy estimation and the closure assumption. *Journal of Applied Ecology* **46**, 1173-1181 (2009).

52. C. L. Outhwaite *et al.*, Prior specification in Bayesian occupancy modelling improves analysis of species occurrence data. *Ecological Indicators* **93**, 333-343 (2018).

53. M. Plummer, "JAGS: A program for analysis of Bayesian graphical models using Gibbs sampling". in Proceedings of the 3rd International Workshop on Distributed Statistical Computing (Vienna, Austria, 2003), pp. 1-10.

54. K. Kellner, jagsUI: a wrapper around rjags to streamline JAGS analyses. R package version. 2015;1(1). (2015).

55. M. Kéry, J. A. Royle, *Applied Hierarchical Modeling in Ecology: Analysis of Distribution, Abundance and Species Richness in R and BUGS: Volume 1: Prelude and Static Models* (Academic Press, 2015).

56. K. E. Jones *et al.*, PanTHERIA: a species-level database of life history, ecology, and geography of extant and recently extinct mammals. *Ecology* **90**, 2648-2648 (2009).

57. S. A. Thornton, (Un)tangling the Net, Tackling the Scales and Learning to Fish: An Interdisciplinary Study in Indonesian Borneo. (University of Leicester, Leicester, 2017), 371 pp.

58. S. A. Thornton *et al.*, Towards biocultural approaches to peatland conservation: The case for fish and livelihoods in Indonesia. *Environmental Science & Policy* **114**, 341-351 (2020).
